# A common pathomechanism in GMAP-210– and LBR-related diseases

**DOI:** 10.1172/jci.insight.121150

**Published:** 2018-12-06

**Authors:** Anika Wehrle, Tomasz M. Witkos, Judith C. Schneider, Anselm Hoppmann, Sidney Behringer, Anna Köttgen, Mariet Elting, Jürgen Spranger, Martin Lowe, Ekkehart Lausch

**Affiliations:** 1 Department of Pediatrics, Medical Centre-University of Freiburg, Faculty of Medicine, University of Freiburg, Freiburg, Germany.; 2 Faculty of Biology, Medicine and Health, University of Manchester, Manchester, United Kingdom.; 3 Institute of Genetic Epidemiology, Medical Centre-University of Freiburg, Faculty of Medicine, University of Freiburg, Freiburg, Germany.; 4 Department of Clinical Genetics, VU University Medical Center, Amsterdam, Netherlands.

**Keywords:** Cell Biology, Genetics, Genetic diseases, Protein traffic

## Abstract

Biallelic loss-of-function mutations in *TRIP11*, encoding the golgin GMAP-210, cause the lethal human chondrodysplasia achondrogenesis 1A (ACG1A). We now find that a homozygous splice-site mutation of the lamin B receptor (*LBR*) gene results in the same phenotype. Intrigued by the genetic heterogeneity, we compared GMAP-210– and LBR-deficient primary cells to unravel how particular mutations in *LBR* cause a phenocopy of ACG1A. We could exclude a regulatory interaction between LBR and GMAP-210 in patients’ cells. However, we discovered a common disruption of Golgi apparatus architecture that was accompanied by decreased secretory trafficking in both cases. Deficiency of Golgi-dependent glycan processing indicated a similar downstream effect of the disease-causing mutations upon Golgi function. Unexpectedly, our results thus point to a common pathogenic mechanism in GMAP-210– and LBR-related diseases attributable to defective secretory trafficking at the Golgi apparatus.

## Abstract 2

Mutations in lamin B receptor or TRIP11 cause a common clinical and cellular phenotype attributable to impaired secretory trafficking and aberrant glycan processing by the Golgi apparatus.

## Graphical abstract

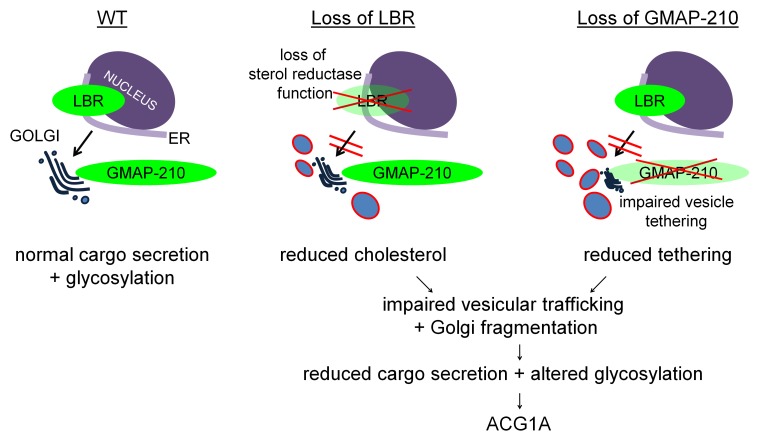

## Introduction

Recessive loss-of-function mutations in the lamin B receptor (*LBR*) and the thyroid hormone receptor interactor 11 (*TRIP11*) genes cause lethal human chondrodysplasias with distinct phenotypes (1, 2). Owing to the dual functions of the LBR protein, which acts as both a receptor for lamin B and an enzyme of the cholesterol biosynthesis pathway, LBR-linked diseases are currently classified as laminopathies as well as disorders of cholesterol biosynthesis (3, 4). There is a remarkable genotype-phenotype correlation; depending on the state, nature, and site of mutation, the phenotypes range from asymptomatic to embryonic lethal (3, 5). In a heterozygous state, some, but not all, *LBR* mutations cause Pelger-Huët anomaly (PHA, MIM 169400), a benign condition just marked by the hypolobulation of granulocyte nuclei (5). Compound heterozygous *LBR* mutations were recently identified in PHA cases affected by mild skeletal anomalies and short stature (6, 7). Furthermore, an intermediate phenotype, displaying mental retardation and skeletal defects, was also described among the original PHA cases. There, a homozygous splice acceptor mutation leading to the skipping of exon 13 of *LBR* was found (8). At the severe end, biallelic loss-of-function mutations of *LBR* fatally disrupt skeletal development, resulting in the clearly recognizable clinical entity of hydrops-ectopic-calcification-moth-eaten (HEM), also known as Greenberg dysplasia (GRBGD, MIM 215140) (9). Interestingly, the very same heterozygous mutations found in PHA lead to GRBGD when both alleles are affected (3, 10). GRBGD is currently included in the chondrodysplasia punctata (CDP) group of diseases, which covers heterogeneous metabolic skeletal dysplasias with overlapping clinical features (2). Enzyme deficiencies in CDP are believed to converge on lipid biogenesis (11). Notably, similar to GRBGD, enzymes of cholesterol metabolism are also deficient in a number of CDP types. In keeping with a previous report of abnormal cholesterol metabolites in fetal GRBGD tissues (10), this finding argues in favor of GRBGD being in fact an inborn error of metabolism, the more so as recent data indicate that disease-associated LBR variants have reduced 3β-hydroxysterol Δ^14^ (C14 sterol) reductase activity (12, 13). However, there is no genuine in vivo model faithfully recapitulating LBR-associated human disease (14–16). Hence, the relevance of these findings for disease development remains presently unclear, as all functional studies to date have been conducted in heterologous tumor cell lines (4, 14). The pathology of bone lesions in other CDP types is unknown as well, so this does not help in the determination of potential disease mechanisms in GRBGD (11). Therefore, despite some evidence for a physiological role of LBR in sterol biogenesis and inner nuclear membrane integrity, the pathophysiology of GRBGD and other LBR-related diseases remains largely obscure.

We report here on a case carrying what we believe is a novel homozygous splice site mutation of *LBR*. This mutation is predicted to cause a complete abrogation of both the lamina-binding function and the cholesterol reductase activity of LBR. On the basis of current knowledge, a recessive loss-of-function mutation of *LBR* would be expected to cause GRBGD. On the contrary, hallmarks of GRBGD, such as a moth-eaten radiographic appearance of the long bones and ectopic calcification, were lacking in our case. Instead, the phenotype fully matched that of achondrogenesis 1A (ACG1A, MIM 200600), and thereby represents to our knowledge the first instance of this disease caused by mutations in *LBR*. ACG1A is considered to be one of the most severe human chondrodysplasias and has a specific clinical presentation (1). Since all reported cases of ACG1A to date are due to mutations in *TRIP11* (17, 18), which encodes a golgin also known as GMAP-210 (Golgi-microtubule-associated protein, 210 kDa), we were curious to find out whether the striking clinical similarity of *LBR*- and *TRIP11*-associated achondrogenesis point to a common Golgi-related pathophysiology. Golgins are coiled-coil proteins that mediate vesicle tethering at the Golgi complex important for vesicular trafficking and Golgi organization (19, 20). However, there is conflicting evidence about the role of GMAP-210 in secretory protein transport (21–24). We therefore investigated the molecular consequences of disease-causing mutations in ACG1A-derived primary cells and found that not only GMAP-210, but also LBR, are indeed both required for efficient protein trafficking and processing in the secretory pathway. As secretory trafficking and cargo processing are of particular importance for extracellular matrix production by chondrocytes, our results can explain the developmental skeletal defects seen in ACG1A and in diseases caused by LBR deficiency.

## Results

### LBR-null mutations cause a phenocopy of ACG1A.

**Figure 1:**
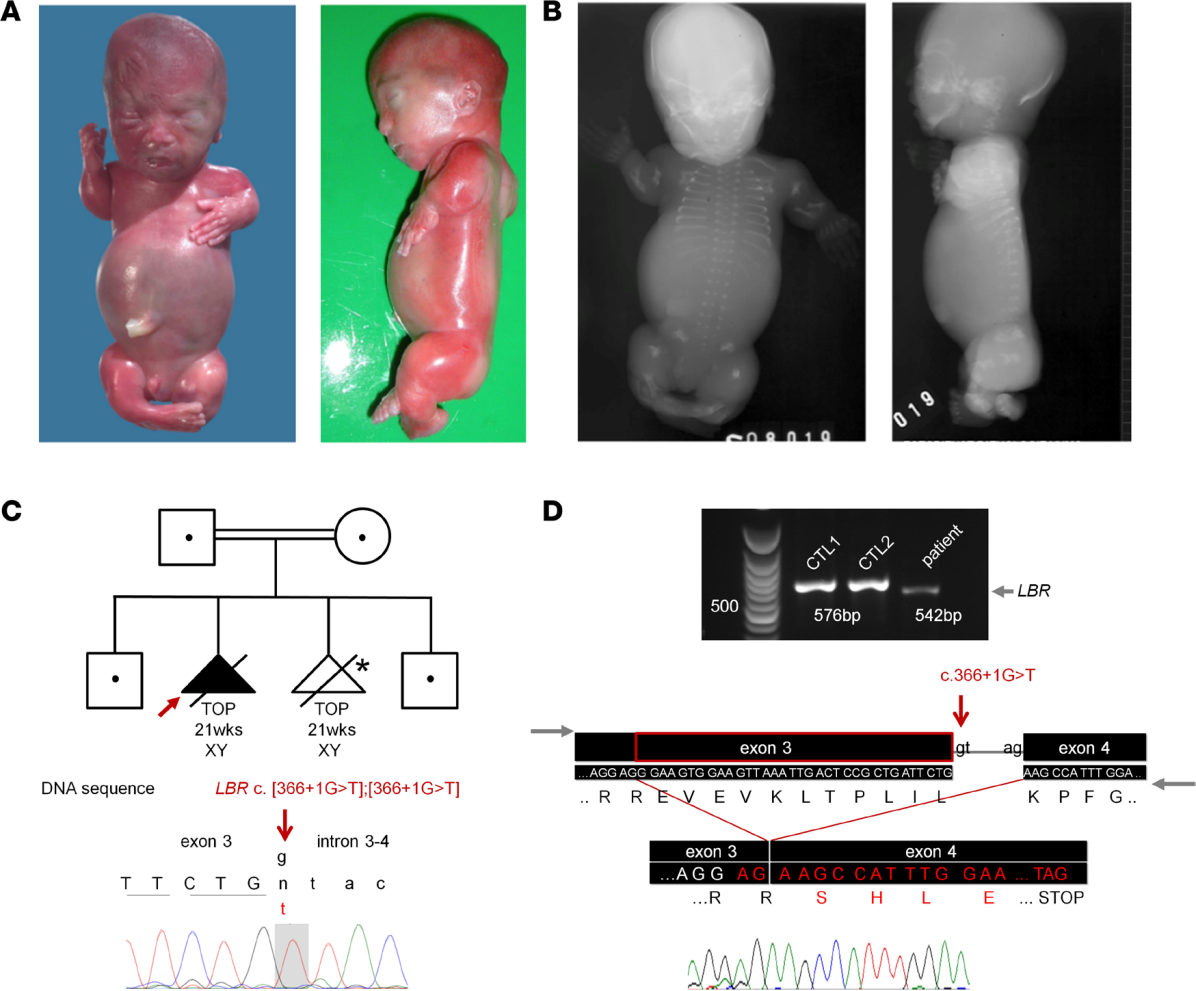
*LBR* mutations cause a phenocopy of achondrogenesis 1A (ACG1A). (**A**) Clinical photographs of the fetus at 21 weeks and 5 days, presenting with hydrops, shortened extremities, protruding abdomen, and short trunk. (**B**) Radiographs in frontal and lateral view show delayed ossification of spine and pelvis. Note the barrel-shaped thorax with horizontally oriented ribs and the extremely shortened long bones. (**C**) Sanger sequencing of genomic DNA demonstrates a homozygous mutation of the *LBR* gene (c.[366+1g>t];[366+1g>t]) that abrogates the splice donor site of exon 3. The abridged pedigree above shows cosegregation of the *LBR* mutation; *termination of pregnancy (TOP) because of cardiac malformation. (**D**) Exon-spanning reverse transcription PCR analysis of *LBR* (35 cycles) using cDNA derived from patient and matched control fibroblasts (CTL1, 2). Below: Schematic representation and electropherogram of the amplified/sequenced region and the splice mutation of *LBR* (indicated by a red arrow) that causes a partial deletion of exon 3, leading to a frameshift and premature termination codon in the open reading frame of *LBR* (p.E112Sfs*39). Gray horizontal arrows indicate the relative position of primers (not to scale).

We studied a male fetus from the second pregnancy of healthy consanguineous Turkish parents. The pregnancy was terminated at 21 weeks and 5 days because of intrauterine growth retardation and severe micromelia (Figure 1A). In addition, the fetus presented with short neck and trunk, narrow thorax, protuberant abdomen, hydrops, and distinctive radiographic features of ACG1A. Ossification of the skull, rib cage, and limbs was severely reduced. Further, radiographs demonstrated impaired ossification of the spine, the pedicles, and the sacrum. Scapulae were hypoplastic, the ossa ischium and the pubic bones were under-ossified, and an extreme shortening and broadening of the tubular bones was recognized (Figure 1B). Verified by independent experts (European Skeletal Dysplasia Network, http://www.esdn.org) (25), the clinical-radiographic features established a definitive diagnosis of ACG1A.

Sanger sequencing of *TRIP11* failed to provide molecular confirmation for the clinical-radiographic diagnosis. With the assumption of a novel recessive skeletal disease, we used whole exome sequencing (WES) of the index case and his parents for unbiased disease gene identification. Thereby an undescribed homozygous *LBR* c.366+1G>T variant at the boundary of exon 3 was detected. No additional segregating homozygous or heterozygous variants, known or predicted to impair gene function were found by comprehensive bioinformatics analysis. The nucleotide change in *LBR* was validated by Sanger sequencing and cosegregation was confirmed by heterozygosity of the unaffected parents. Unaffected siblings were either carriers or wild type (Figure 1C). The variant was neither listed as a single nucleotide polymorphism (SNP) in public databases, nor found in an in-house exome database of matched ethnicity. In silico analysis predicted aberrant splicing with activation of an ectopic splice site within exon 3 (26).

**Figure 2:**
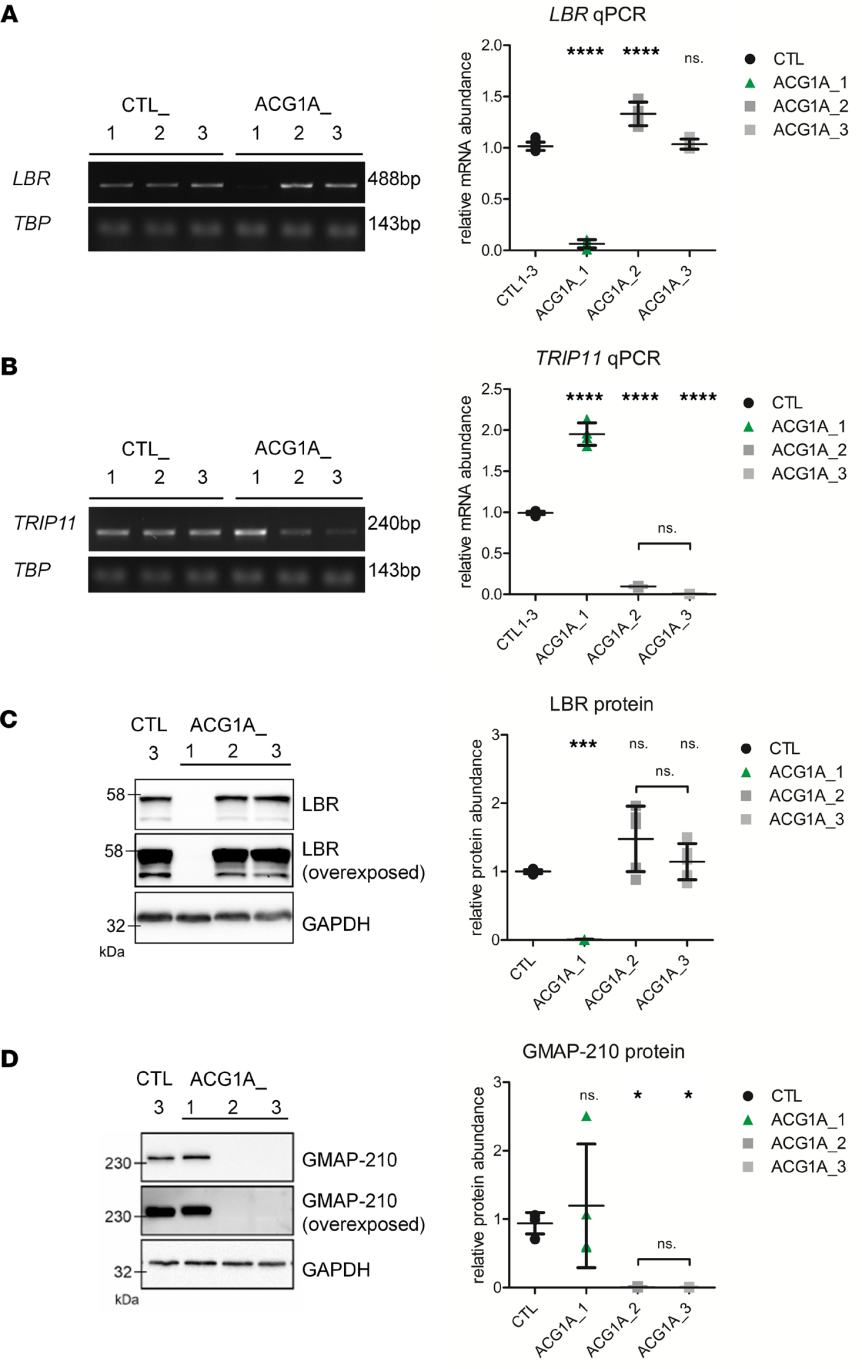
Analysis of *LBR* and *TRIP11* mRNA and protein expression in achondrogenesis 1A (ACG1A) patient–derived primary cells. Semiquantitative reverse transcription PCR (left, 25 cycles), and quantitative real-time PCR (RT-qPCR) analysis (right) of (**A**) *LBR,* and (**B**) *TRIP11* of cDNA derived from patient and control primary fibroblasts. ACG1A_1 carries the homozygous *LBR* mutation described in this study, ACG1A_2 and 3 bear biallelic mutations of *TRIP11*; CTL_1 to 3 are matched wild-type controls. *TBP* was used for normalization; the same agarose gel is shown in **A** and **B**, which are representative of 3 independent experiments (*N* = 3). For RT-qPCR results, the average value of the controls was set to 1 (*n* = 12). Horizontal lines represent the mean of quadruplicates (*n* = 4), error bars indicate SD. Statistical differences were assessed by 1-way ANOVA using Bonferroni’s correction for multiple comparisons; ns., not significant. ****P < 0.0001. Immunoblots of (**C**) LBR and (**D**) GMAP-210 protein in whole-cell lysates of patients and controls; GAPDH staining indicates total protein loading and was used for normalization in quantitative signal analysis (right). Horizontal lines represent the mean of triplicate quantitative blot signal analyses (*n* = 3), error bars indicate SD. Statistical differences were assessed by 1-way ANOVA using Bonferroni’s correction for multiple comparisons; ns., not significant. *P ≤ 0.05, ***P < 0.001. See complete unedited blots in the supplemental material.

To verify an effect on splicing, cDNA derived from primary fibroblasts of the fetus was analyzed by PCR with primers spanning the region of interest. We thereby identified a smaller amplicon of 542 bp instead of 576 bp expected for wild-type *LBR* (Figure 1D). Sequencing of the PCR product implied the activation of an ectopic donor site, resulting in partial loss of exon 3 of *LBR*. In consequence, a frameshift leads to a premature translational stop codon in the open reading frame (ORF) of mutant *LBR* (LBR p.E111Sfs*39) that most likely induces nonsense-mediated mRNA decay (NMD) (27). In accordance, only a faint *LBR*-specific signal was seen using nonquantitative RT-PCR analysis. *LBR* mRNA abundance was strongly reduced in primary fibroblasts obtained from the fetus (in the following referred to as ACG1A_1) (Figure 1D, Figure 2A, and Table 1). The early stop codon generated by the c.366+1G>T mutation predicts a truncated, nonfunctional LBR protein. Accordingly, no LBR protein was detectable by immunoblotting (Figure 2C), not even by overexposure of the membrane. This is in contrast to GRBGD, where residual aberrant LBR proteins comprising the lamina-binding domain are found (12). Thus, to the best of our knowledge our case represents the first true LBR-null phenotype.

### There is no regulatory interaction between LBR and GMAP-210.

Since we had verified the *LBR* mutation to be disease-causing in *TRIP11*-negative ACG1A, we were intrigued to unravel the pathophysiological basis of genetic heterogeneity. We first investigated whether a loss of *LBR* had an effect on *TRIP11* abundance. For this purpose, we compared primary LBR-deficient fetal fibroblasts (ACG1A_1) to fibroblasts of ACG1A cases with known *TRIP11* nonsense mutations (ACG1A_2 and 3; Table 1) (17). Matched fibroblasts of healthy donors without mutations in *TRIP11* and/or *LBR* served as controls (CTL 1 to 3). Interestingly, the *TRIP11* mRNA concentration was approximately 2-fold higher in LBR-deficient cells (Figure 2B). However, since the amount of GMAP-210 protein was the same as in wild-type control (Figure 2D), we did not assume a significant effect ensuing from this upregulation. Vice versa, biallelic nonsense mutations of *TRIP11* caused a strong reduction of *TRIP11* mRNA and a complete loss of the GMAP-210 protein (Figure 2, B and D), while *LBR* expression levels remained unaffected (Figure 2, A and C).

**Figure 3:**
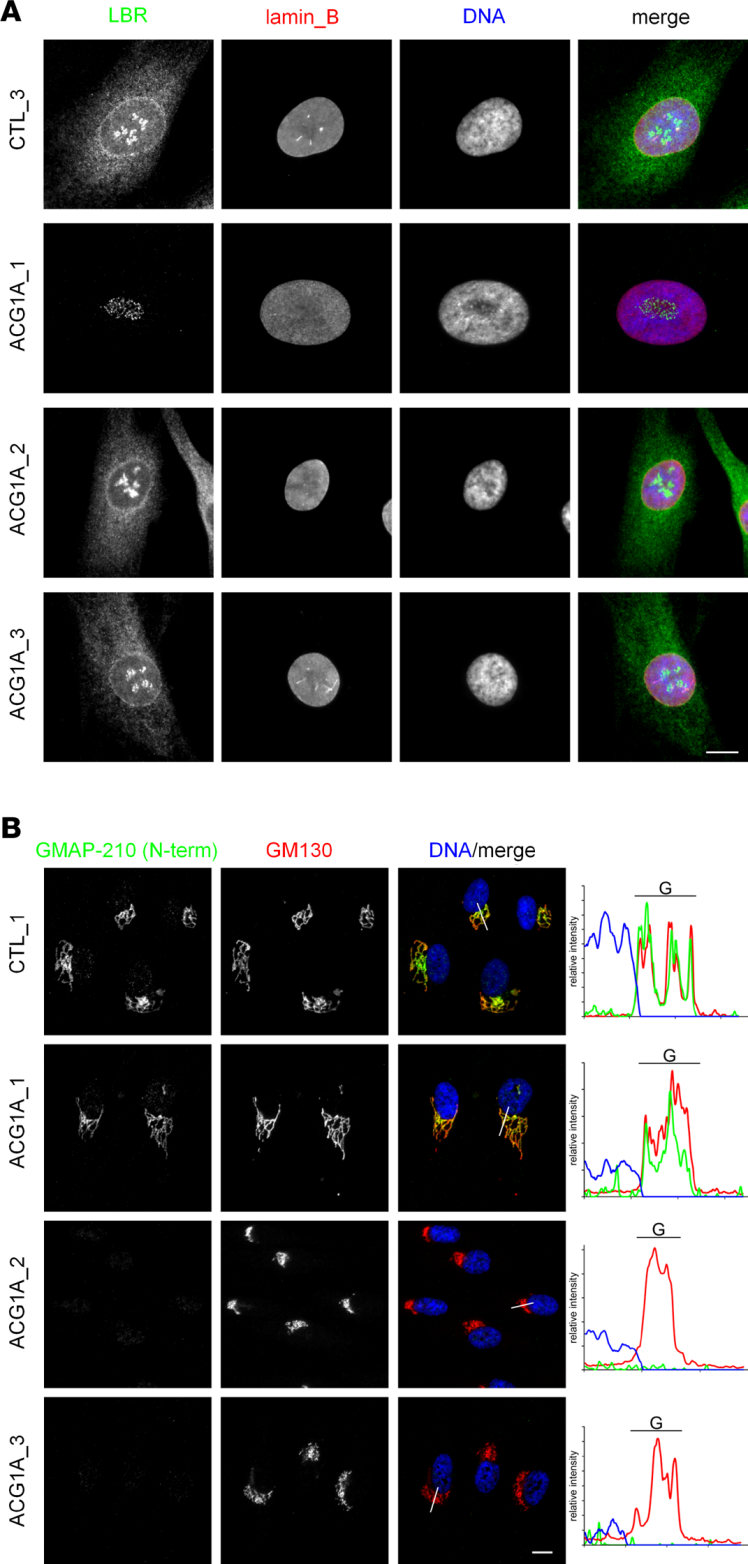
Loss of LBR or GMAP-210 in achondrogenesis 1A (ACG1A) patient–derived primary cells. Immunofluorescence microscopy of patient and control fibroblasts; ACG1A_1 carries the homozygous *LBR* mutation described in this study, ACG1A_2 and 3 bear biallelic mutations of *TRIP11*; CTL_1 and 3 are representative matched wild-type controls. Cells were grown on coverslips, fixed, and costained with (**A**) LBR- and lamin B–specific antibodies, or (**B**) using antibodies directed against GMAP-210 and the *cis*-Golgi marker GM130. Nuclear DNA was stained with Hoechst 33342 dye. White lines indicate where red, green, and blue (RGB) fluorescence was quantified; RGB profile plots of the sections are shown on the right. Scale bars: 10 μm.

We next analyzed the intracellular distribution and abundance of LBR and GMAP-210 proteins by immunofluorescence microscopy. In keeping with previous reports (12, 13), control and GMAP-210–deficient cells showed a strong signal of the LBR confined to the nuclear envelope (Figure 3A). Less intense staining was detected in the nuclear matrix, in the nucleoli, and throughout the endoplasmic reticulum (ER). In contrast, LBR staining was absent from the fibroblasts of ACG1A_1, confirming the loss of the LBR protein and the specificity of the antibody (Figure 3A). Lack of LBR did not change the intracellular localization of GMAP-210, as the intense *cis*-Golgi–specific staining in ACG1A_1 cells was not different from controls (Figure 3B). As expected, no GMAP-210 signal was detected in the 2 ACG1A cases bearing biallelic nonsense mutations of *TRIP11* (Figure 3B). Importantly, *cis*-Golgi staining by anti-GM130 antibodies revealed that the absence of GMAP-210 results in severe compaction of the Golgi apparatus, as reported previously (28) for transient knockdown experiments in generic cell lines (Figure 3B). To the best of our knowledge, this study is the first to investigate the consequences of disease-specific *TRIP11* mutations in patient-derived primary cells. Our observations support the view that GMAP-210 is an important structural component of the Golgi complex in humans (23). Taken together, our results show that loss-of-function mutations of *LBR* had no apparent effect on GMAP-210, thereby excluding the possibility that the associated ACG1A phenotype is due to a downregulation or mislocalization of GMAP-210.

### LBR-deficient cells are sensitive to cholesterol restriction.

**Figure 4:**
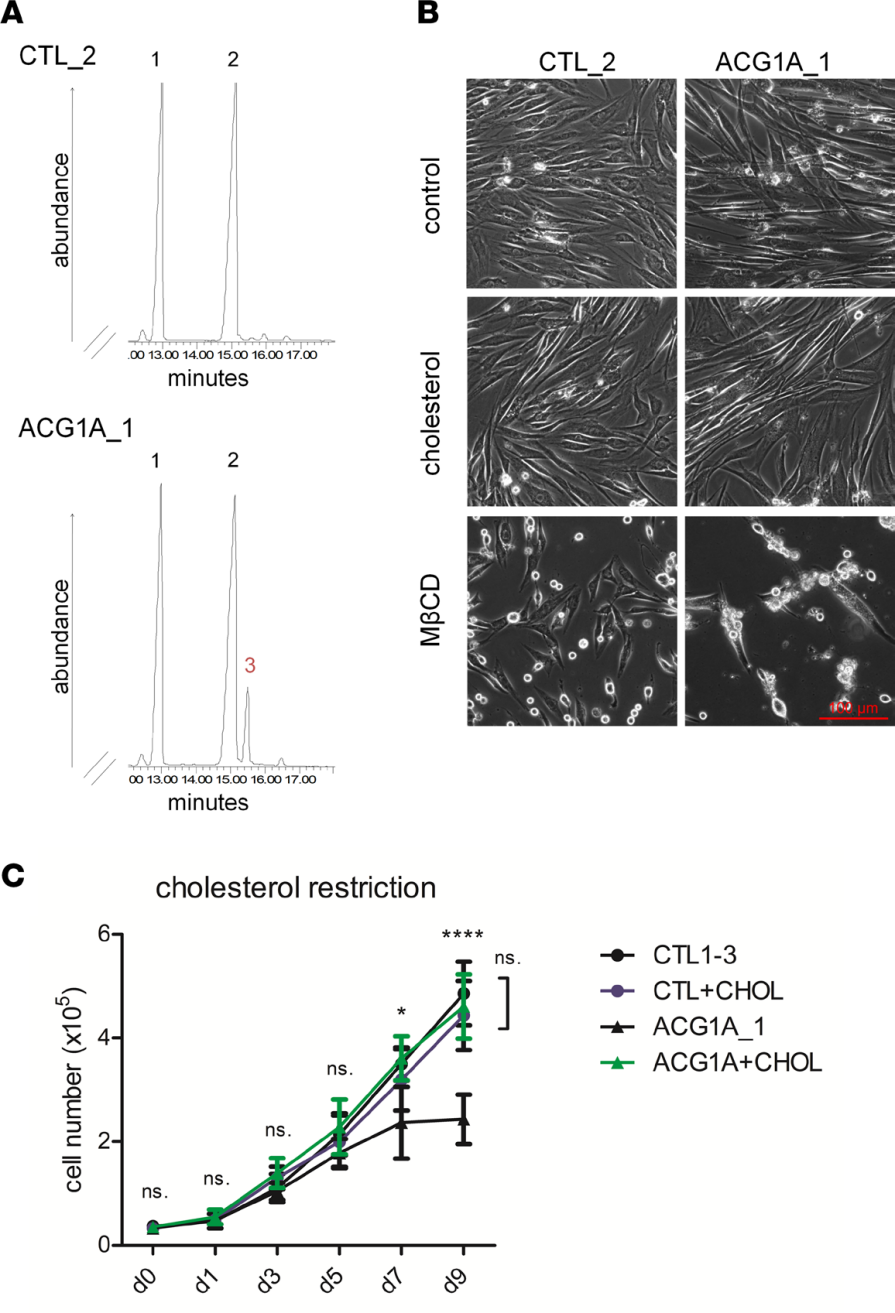
C14 sterol reductase activity is impaired in LBR-deficient cells. (**A**) LBR-deficient primary fibroblasts and controls were cultured in lipid-depleted medium for 5 days, and total lipids were extracted and analyzed by gas chromatography/mass spectrometry. ACG1A_1 carries the homozygous *LBR* mutation described in this study; CTL_2 is representative of 3 matched wild-type controls (*n* = 3). Peak 1: 5β-cholestan-3α-ol (internal standard). Peak 2: cholesterol. Peak 3 (red number) was only detected in ACG1A_1 and corresponds to 8,14-cholestadien-3β-ol previously described in Greenberg dysplasia. The data are representative of 3 independent experiments (*n* = 3). (**B**) Indicated primary cells were cultured under cholesterol-restrictive growth conditions for 2 days, the medium was supplemented either with 2.5 mM methyl-β-cyclodextrin (MβCD, cholesterol depletion), or with 2.5 mM MβCD:cholesterol-saturated complexes (cholesterol replacement), and imaged by bright-field microscopy. The data are representative of 3 experiments (*n* = 3). (**C**) Growth curves of LBR-deficient cells and 3 matched controls (*n* = 3) in lipid-depleted medium for 9 days; 6.5 μM cholesterol was added to the medium of ACG1A_1 (ACG1A_1+CHOL) and 3 controls (CTL+CHOL). Cell numbers were determined by automated counting; data points represent the mean of triplicates values; for controls the mean of 3 pooled triplicates was determined (*n* = 9); error bars indicate SD. Statistical differences were assessed by 2-way ANOVA using Bonferroni’s correction for multiple comparisons; ns., not significant. *P ≤ 0.05, ****P < 0.0001.

Because GRGBD-associated mutations inactivate the C14 sterol reductase domain of LBR and lead to an intracellular accumulation of precursors in the cholesterol biosynthesis pathway (10, 13, 29), we analyzed the sterol composition of ACG1A_1 cells grown in lipoprotein-depleted medium. As described previously for GRGBD (10), gas chromatography/mass spectrometry (GC/MS) analysis revealed the cholesterol precursor cholesta-8,14-dien-3β-ol in ACG1A_1 cells that was not detectable in matched healthy controls (Figure 4A). We thus concluded that both GRBGD- and ACG1A-associated *LBR* mutations cause a loss of sterol reductase function.

As C14 sterol reductase deficiency is known to impair de novo sterol biosynthesis in humans (12, 13), we further investigated whether ACG1A_1 cells are auxotrophic and depend on the delivery of exogenous cholesterol for growth and viability. Cellular cholesterol levels can be specifically manipulated by the addition of cyclodextrin complexes to conditioned media (30). The high affinity of cyclodextrins for cholesterol can be not only used to remove cholesterol from biological membranes, but also to generate cholesterol inclusion complexes that donate cholesterol and thereby increase cellular cholesterol levels (31). A complete knockout of *LBR* has been shown to decrease viability of HeLa cells under cholesterol-restrictive growth conditions (12). In order to determine if C14 sterol reductase–deficient primary cells are auxotrophic as well, ACG1A_1 and control cells were grown for 48 hours in serum-free DMEM supplemented either with methyl-β-cyclodextrin (MβCD) to deplete cholesterol, or with MβCD:cholesterol complexes to replicate cholesterol-proficient conditions in vivo. Compared with control cells, we observed a higher number of ACG1A_1 fibroblasts exhibiting cell rounding and detachment under cholesterol depletion, which indicates a higher susceptibility to cholesterol restriction (Figure 4B). Accordingly, addition of MβCD:cholesterol–saturated complexes to the medium rescued the observed phenotype in ACG1A_1 cells (Figure 4B). LBR was also rate limiting for proliferation under cholesterol-restrictive conditions. While controls showed exponential growth in lipid-depleted medium, the growth rate of ACG1A_1 cells progressively declined to zero from days 5 to 9, consistent with growth arrest (Figure 4, C and D). Importantly, proliferation and viability were restored by the addition of cholesterol to the medium of ACG1A_1 cells. Hence, ACG1A-associated *LBR* mutations cause an error of metabolism characterized by intracellular accumulation of unreduced sterol derivatives and impaired de novo synthesis of cholesterol, which reduces cell proliferation and viability.

### Nuclear envelope integrity is preserved in LBR-mutant human primary cells.

To analyze intracellular compartments that may be affected in ACG1A, we performed high-resolution imaging studies, focusing on the nucleus, the ER, and the Golgi apparatus as the key intracellular locations of the LBR and GMAP-210 proteins. LBR has long been implicated in nuclear envelope integrity and anchoring to the nuclear lamina, as documented by *LBR* mutations leading to nuclear hyposegmentation of granulocytes (Pelger-Huët anomaly) (32–35). The nuclear envelope comprises outer and inner membranes that enclose the luminal space. The inner nuclear membrane is lined with lamins and lamin-binding membrane proteins, such as LBR, which constitute the lamina (3, 34, 36). The lamina connects to the nucleoplasm and controls critical nuclear functions, including mitosis, meiosis, and apoptosis, but also chromatin conformation and thereby transcriptional activity (4). A laminar defect would therefore likely have severe consequences for basic cellular functions and is believed to be pathogenic in GRBGD (4).

However, alterations of the nuclear shape and envelope were neither observed in granulocytes of a fetal blood smear collected upon autopsy, nor in ACG1A_1 fetal fibroblasts by transmission electron microscopy (TEM) (Supplemental Figure 1A; supplemental material available online with this article; https://doi.org/10.1172/jci.insight.121150DS1). As expected, a regular nuclear morphology was detected in *TRIP11*-mutant cells as well (Supplemental Figure 1A). In all analyzed cells, both nuclear membranes appeared correctly folded, and the ultrastructure of the luminal space and the intermembrane distance between the inner and the outer membrane were normal (Supplemental Figure 1B). This is in line with a recent in-depth analysis of inner nuclear membrane proteins and components of the nuclear lamina in *LBR*-knockout HeLa cells, which showed no effect on the localization of structural proteins of the nuclear envelope and other nuclear markers, and no laminar defects (12). Although we cannot exclude laminar alterations in other cell types, our results in primary fibroblasts provide no evidence that a loss of the lamin-binding function of LBR causes ACG1A, or other developmental anomalies.

### There is no common ER phenotype in ACG1A.

**Figure 5:**
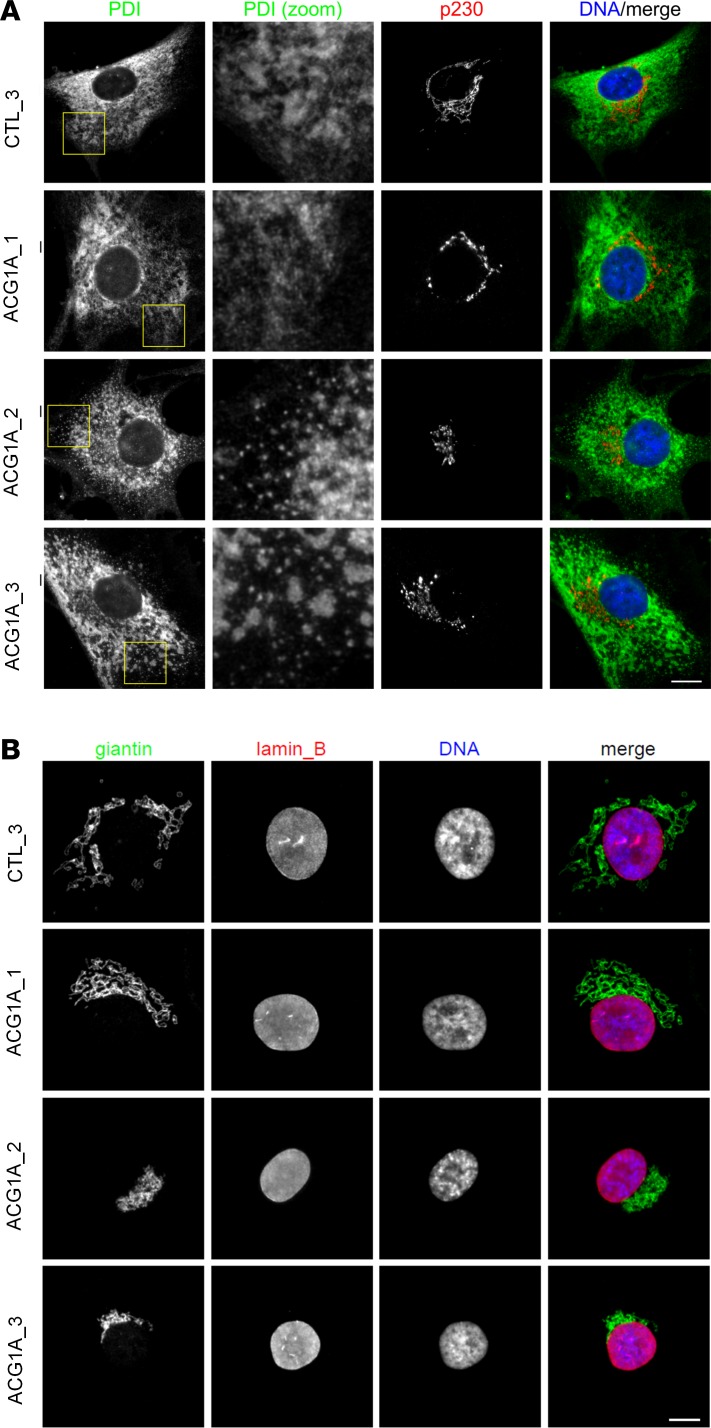
Endoplasmic reticulum (ER) and Golgi morphology in achondrogenesis 1A (ACG1A). Immunofluorescence microscopy of patient and control fibroblasts; ACG1A_1 carries the homozygous *LBR* mutation described in this study, ACG1A_2 and 3 bear biallelic mutations of *TRIP11*; CTL_3 is representative of matched wild-type controls (*n* = 3). Cells were grown on coverslips, fixed, and (**A**) costained with antibodies directed against the ER marker protein disulfide isomerase (PDI) and the *trans*-Golgi protein p230, or (**B**) using antibodies directed against the *cis*- and *medial*-Golgi protein giantin and the LBR ligand lamin B. Nuclear DNA was stained with Hoechst 33342 dye. Scale bars: 10 μm.

Non-nuclear LBR is found in the ER (Figure 3A) where de novo endogenous sterol synthesis takes place (12, 13). In addition to a separation of the inner nuclear membrane from the outer nuclear envelope, mutant variants of LBR induce an expansion of the ER in some cell types (36, 37). GMAP-210 is localized at the ER-Golgi intermediate compartment (ERGIC) and *cis*-Golgi and mediates trafficking processes in the anterograde (ER to Golgi) and retrograde (Golgi to ER) direction (23, 28, 38). As previous ultrastructural analysis of *Trip11*-mutant mice has demonstrated swollen ER cisternae (17), we next investigated whether ACG1A-asssociated mutations may cause a common ER pathology. Protein disulfide isomerase (PDI) is an ER-resident chaperone with disulfide exchange activity (39). We used PDI staining to assess ER morphology in ACG1A. A normal ER structure comprising a mix of ER sheets and tubular structures with the ER-encircling lamina was observed in control and ACG1A_1 cells by immunofluorescence microscopy (Figure 5A). However, higher-resolution imaging using TEM demonstrated a swollen and less reticular ER in cells lacking LBR (Supplemental Figure 2). An irregular ER morphology was also detected by immunofluorescence microscopy in cells lacking GMAP-210, where there appeared to be less continuous ER in the cell periphery, with reduced number of ER sheets and tubules and an increased number of ER foci (Figure 5A, insets). Although these changes may correspond to the ultrastructural changes reported for *Trip11-*mutant mice (17), TEM was not sufficient to confirm comparable structural alterations in fibroblasts. The ER phenotype of *TRIP11*-deficient human cells therefore merits further in-depth investigation.

We also took a closer look at compartments involved in traffic between the ER and the Golgi. Coat protein complex II (COPII) operates in ER exit of cargo and is localized to specialized regions of the ER known as ER exit sites (ERES). In mammalian cells, cargo then passes through the ERGIC en route to the Golgi (40, 41). No substantial differences between patient-derived cells and controls were detected when we analyzed the intracellular distribution of ERGIC53 and SEC13, specific markers of ERGIC and ERES (Supplemental Figure 3). Importantly, in spite of the observed alterations in ER morphology, the abundance and organization of ERES and ERGIC structures was not markedly affected in LBR- or GMAP-210–deficient cells. Taken together, our findings make a common ER-related pathogenic mechanism in ACG1A unlikely. We therefore focused on a possible Golgi phenotype of LBR-mutant cells.

### ACG1A-associated mutations cause common structural alterations of the Golgi apparatus.

**Figure 6:**
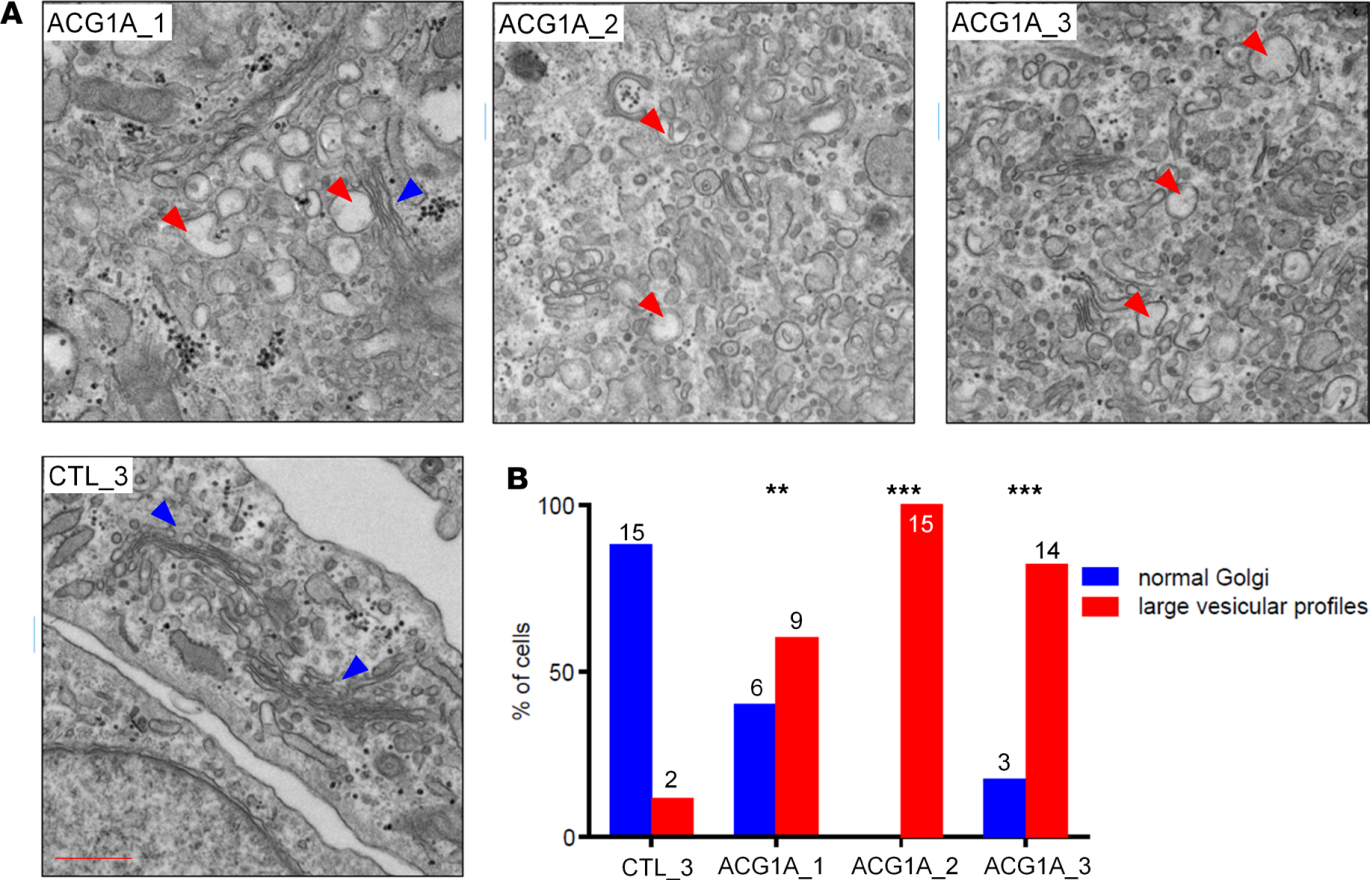
Abnormal vesiculation at the Golgi apparatus is a common cellular phenotype in achondrogenesis 1A (ACG1A). (**A**) Transmission electron microscopy of patient and control fibroblasts; ACG1A_1 carries the homozygous *LBR* mutation described in this study, ACG1A_2 and 3 bear biallelic mutations of *TRIP11*; CTL_3 is representative of 3 matched wild-type controls (*n* = 3). Red arrowheads mark large vesicular profiles, blue arrowheads normal Golgi stacks in ACG1A_1 and control cells. Scale bar: 500 nm. (**B**) Quantification of cells with abnormal vesiculation, defined as peri-Golgi circular profiles with a diameter of >100 nm (red bars) compared with cells with normal Golgi morphology (blue bars); the *y* axis shows the percentage of respective phenotypes, and the individual cell counts are indicated above the bars. The significance of differences in the number of cells with large vesicular profiles was compared using a χ^2^ test; **P < 0.01; ***P < 0.001.

RNAi-mediated depletion of GMAP-210 in generic cell models has been reported to cause Golgi ribbon compaction and loss of Golgi ultrastructure (23), supporting the hypothesis that a Golgi-related pathogenic mechanism underlies ACG1A. This view is further strengthened by findings in *Trip11*-knockout mice, where a disrupted Golgi is observed in certain cell types (17, 42). As expected, mutations of *TRIP11* in patient-derived primary cells caused a disruption of Golgi architecture, with compaction and breaking of the Golgi ribbon (Figure 3B, and Figure 5, A and B). TEM revealed a dramatic loss of cisternal stack architecture and the accumulation of numerous vesicular profiles in the Golgi region (Figure 6, A and B). Although fluorescence microscopy demonstrated no obvious change in Golgi organization in ACG1A_1 cells, where the Golgi ribbon retained its typical appearance (Figure 3B, and Figure 5, A and B), the higher resolution of TEM disclosed a disruption of Golgi ultrastructure similar to that seen upon loss of GMAP-210 (Figure 6, A and B). LBR deficiency led to a marked loss of stacked cisternae and the accumulation of vesicular profiles in the Golgi region. This vesiculation was not seen in matched controls, which exhibited an intact Golgi ultrastructure with clear, stacked cisternae and associated transport vesicles (Figure 6, A and B). Aberrant Golgi vesiculation in ACG1A_1 cells was an unexpected observation, since LBR is strictly confined to other intracellular compartments (Figure 3A) and has no known functional connection to the Golgi. However, it has previously been shown that cellular cholesterol levels are critical for Golgi morphology, and both overloading and depletion may induce vesiculation and dispersal of vesicles (43, 44). Importantly, exogenous cholesterol rescued the structural alterations of the Golgi apparatus in ACG1A_1 cells (Supplemental Figure 4, A and B). Given the enzymatic activity of LBR as a C14 sterol reductase, we therefore assumed that the intracellular accumulation of unreduced cholesterol metabolites [i.e., cholesta-8(9),14-dien-3β-ol and cholesta-8(9),14,24-trien-3β-ol; ref. 10] and/or insufficient de novo cholesterol synthesis in LBR-deficient cells are responsible for the Golgi phenotype (12, 13).

### Secretory traffic and glycan processing by the Golgi complex are impaired in LBR- and GMAP-210–deficient cells.

**Figure 7:**
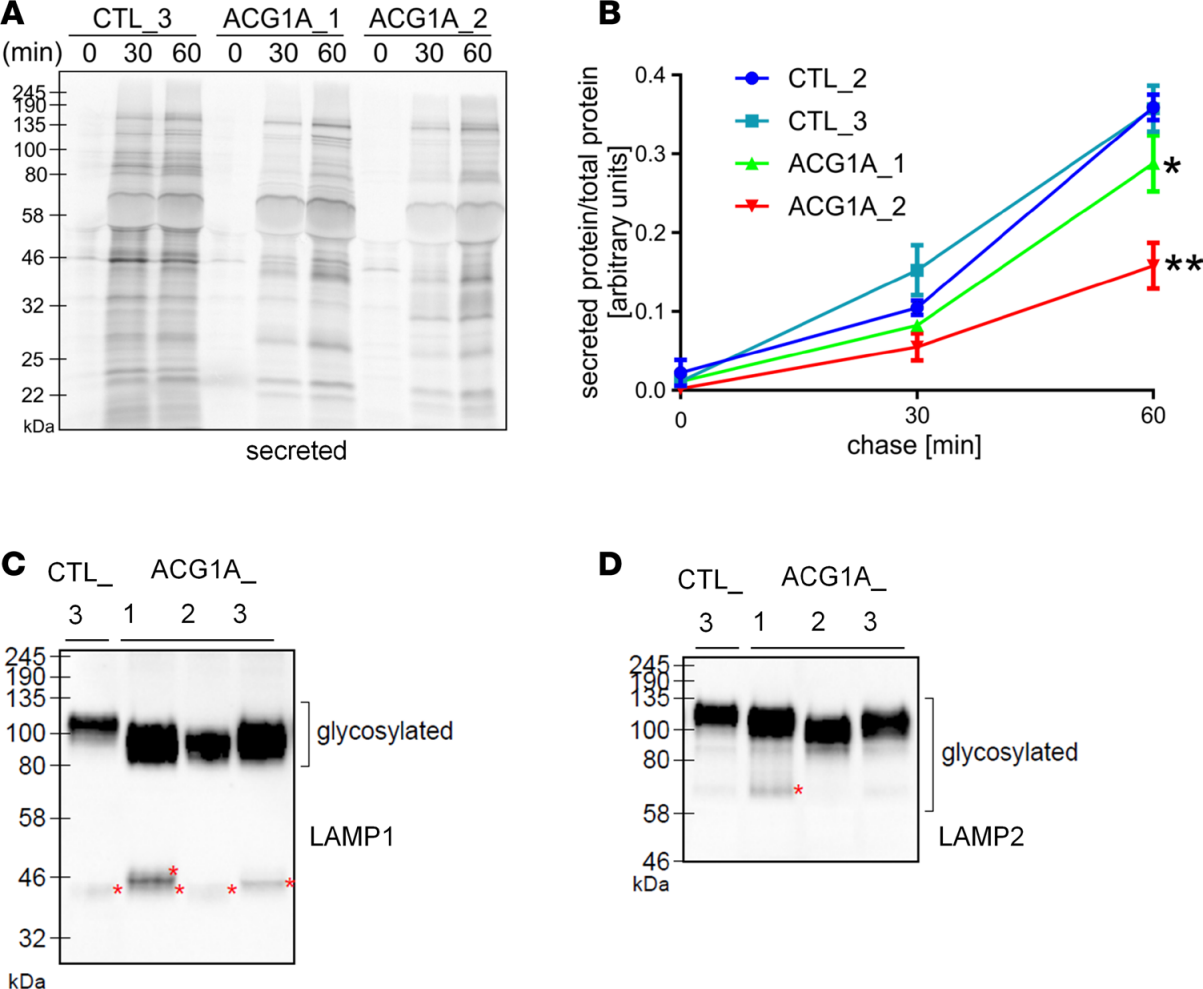
Reduced secretory trafficking and defective glycoprocessing in achondrogenesis 1A (ACG1A). Patient and control fibroblasts were starved for 1 hour in methionine- and cysteine-free medium and pulsed for 20 minutes with fresh starvation medium containing 50 μCi/ml [^35^S]methionine and [^35^S]cysteine protein-labeling mix. Cells were chased in medium containing unlabeled methionine and cysteine for 0, 30, or 60 minutes. Proteins secreted to the medium were precipitated and lysed. (**A**) Radiolabeled secreted proteins in trichloroacetic acid precipitates were subjected to denaturing gel electrophoresis and visualized by phosphorimaging. ACG1A_1 carries the homozygous *LBR* mutation described in this study, ACG1A_2 bears biallelic mutations of *TRIP11*; CTL_2 and 3 are matched wild-type controls (*n* = 2). (**B**) Ratio of secreted-to-total proteins at the indicated time points. Data represent mean ± SEM of 3 independent experiments (*n* = 3; one-way ANOVA with Dunnett’s multiple comparisons test). *P ≤ 0.05, **P < 0.01. (**C**) Immunoblot analyses of LAMP1, and (**D**) LAMP2 proteins in whole-cell lysates of ACG1A patient and control fibroblasts. Membranes were overexposed to detect weaker signals. Low-molecular-weight intermediate glycosylation products of LAMP1 in ACG1A_1 are marked by a red asterisk. Similar, but faster migrating protein species are visible in ACG1A_3 (red asterisk). Non-glycosylated forms are present at very low steady state levels in CTL_3 and ACG1A_2 cells. As for LAMP1, the mature LAMP2 proteins in ACG1A run at a lower molecular weight range than in controls. An intermediate product is present in ACG1A_1 cells (~60 kDa, red asterisk).

We assumed that the severe structural Golgi alterations in ACG1A reflect perturbed secretory trafficking at this organelle. Indeed, RNAi-mediated depletion of GMAP-210 has previously been shown to reduce secretory traffic in human cells (23). To measure bulk secretory protein trafficking in ACG1A patient–derived primary cells, we used a pulse-chase approach with radiolabeled amino acids (23, 45). Secretion of newly synthesized proteins into the extracellular space was significantly reduced in GMAP-210–deficient cells, indicating a delay in secretory protein transport to the plasma membrane (Figure 7, A and B, and Supplemental Figure 5). Reduced secretion was also observed in ACG1A_1 fibroblasts, albeit to a lesser extent than in GMAP-210–deficient cells (Figure 7, A and B). Interestingly, the profiles of secreted proteins differed between controls and ACG1A, but also slightly between GMAP-210– and LBR-deficient cells (Figure 7A). Thus, the trafficking defect may affect cargo proteins differently, with considerable overlap in ACG1A.

Golgi trafficking ensures correct posttranslational modification of proteins, and disruption of this process often manifests as altered cargo protein glycosylation. Loss of Gmap-210 in mouse fibroblasts has previously been shown to impair glycosylation of specific cargoes (17). We therefore analyzed glycan processing in ACG1A patient cells by means of lysosome-associated membrane proteins (LAMPs), which are sensitive indicators of Golgi glycosylation capacity. LAMPs are lysosomal transmembrane proteins that undergo extensive glycosylation by the traditional N- and O-linked ER-to-Golgi glycan biosynthesis pathways (46). In addition, several of the N-linked adducts are modified by long poly-N-acetyl-lactosamines added exclusively in the Golgi apparatus (46). The molecular mass of the polypeptide backbone of LAMP1 and LAMP2 is approximately 40–45 kDa and increases 3-fold to approximately 120 kDa after glycosylation (47). Glycosylation of LAMP1 and LAMP2 was assessed by Western blot, and demonstrated aberrant electrophoretic mobility patterns in all ACG1A cells (Figure 7, C and D). As compared with wild-type cells, a lower average molecular weight range indicated hypoglycosylated species of both LAMP proteins in mutant primary fibroblasts, consistent with an incomplete modification of proteins in the Golgi. Further, we recognized additional low-molecular-weight, immature LAMP species in LBR- and GMAP-210–deficient cells, which are normally not detectable because they consist of short-lived intermediate products synthesized in the ER (Figure 7, C and D) (48). Notably, we also detected an increase in hypoglycosylated immature LAMPs when cholesterol was depleted from wild-type control cells by MβCD (Supplemental Figure 6A) (30). Vice versa, supply of exogenous cholesterol to lipoprotein-starved ACG1A_1 cultures restored the glycosylation of immature LAMP species (Supplemental Figure 6B).

**Figure 8:**
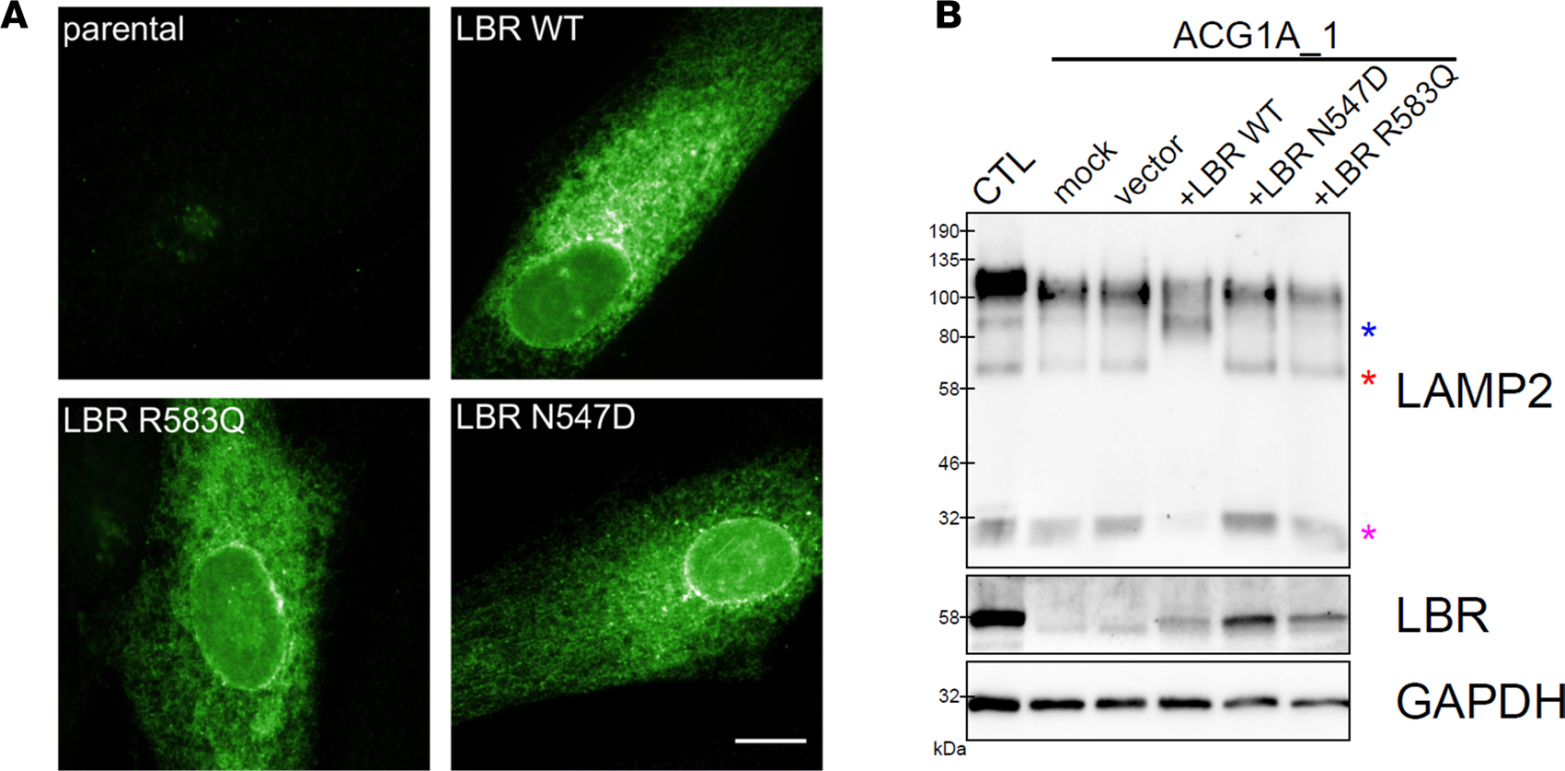
LBR sterol reductase activity restores glycan processing in LBR-deficient cells. (**A**) Immunofluorescence microscopy of ACG1A_1 fibroblasts stained with LBR-specific antibodies; ACG1A_1 carries the homozygous *LBR*-null mutation. Cells were either not transfected (parental) or transiently transfected with vectors overexpressing the indicated LBR variants; the Greenberg dysplasia–associated p.N547D and p.R583Q missense mutations specifically abrogate C14 sterol reductase activity but do not alter the chromatin/lamin-binding properties of the respective LBR protein. WT, wild type. Scale bar: 10 μm. (**B**) ACG1A_1 cells were either electroporated without plasmid (mock), with empty vector (vector), or with expression constructs encoding the indicated LBR proteins. Whole-cell protein lysates of nontransfected wild-type controls (CTL) and ACG1A_1 transfectants were obtained 96 hours after electroporation and analyzed by Western blotting with LBR- and LAMP2-specific antibodies. GAPDH immunostaining of the stripped membrane indicates total protein loading. Fast-migrating nonglycosylated LAMP2 is highlighted by a pink asterisk at approximately 30 kDa; intermediate glycosylation products are marked by a red asterisk at approximately 65 kDa. In ACG1A_1 cells overexpressing wild-type LBR, nonglycosylated LAMP2 and intermediate glycosylation products are not detectable and mature LAMP2 has a similar molecular weight to that in nontransfected wild-type fibroblasts with an additional intermediate band at approximately 85 kDa (blue asterisk), likely corresponding to a more highly modified species than that seen upon LBR deficiency, although not fully mature. Note that a higher protein load of nontransfected wild-type fibroblasts was used to visualize the normal electrophoretic mobility pattern of LAMP2 species.

To corroborate that a functional Golgi defect in LBR-deficient cells solely results from the loss of sterol reductase activity of the protein, we next transiently overexpressed different LBR constructs in ACG1A_1 cells. The GRBGD-associated LBR mutations p.N547D and p.R583Q substitute amino acids highly conserved in homologous C14 sterol reductases and have recently been shown to selectively abrogate the binding capacity of the enzymatic core for reduced nicotinamide adenine dinucleotide phosphate (NADPH), a cofactor absolutely required for the reduction of sterol substrates by LBR (12). While the GRBGD-associated LBR missense mutants are hence enzymatically dead, the other functions and properties of the protein remain unaffected, in particular the lamin/chromatin interaction (12, 13). Accordingly, all exogenous LBR proteins transiently overexpressed in ACG1A_1 cells showed similar abundance and the same subcellular distribution as the endogenous protein in wild-type fibroblasts (Figure 3A), with a strong signal confined to the nuclear envelope and less intense staining of the nuclear matrix, the nucleoli, and throughout the ER (Figure 8A). Despite their correct intracellular localization, enzymatically inactive LBR mutants failed to restore normal glycan processing. In contrast, overexpression of wild-type LBR resulted in a strong reduction of immature LAMP2 species. Importantly, only the restitution of enzymatically competent LBR shifted high-molecular-weight LAMP2 species in ACG1A_1 towards the larger forms seen in wild-type fibroblasts, indicating normal protein maturation by Golgi-resident glycosyltranferases (Figure 8B). Thus, rescue of the functional Golgi defect in LBR-deficient cells is exclusively mediated by the C14 sterol reductase function of LBR.

**Figure 9:**
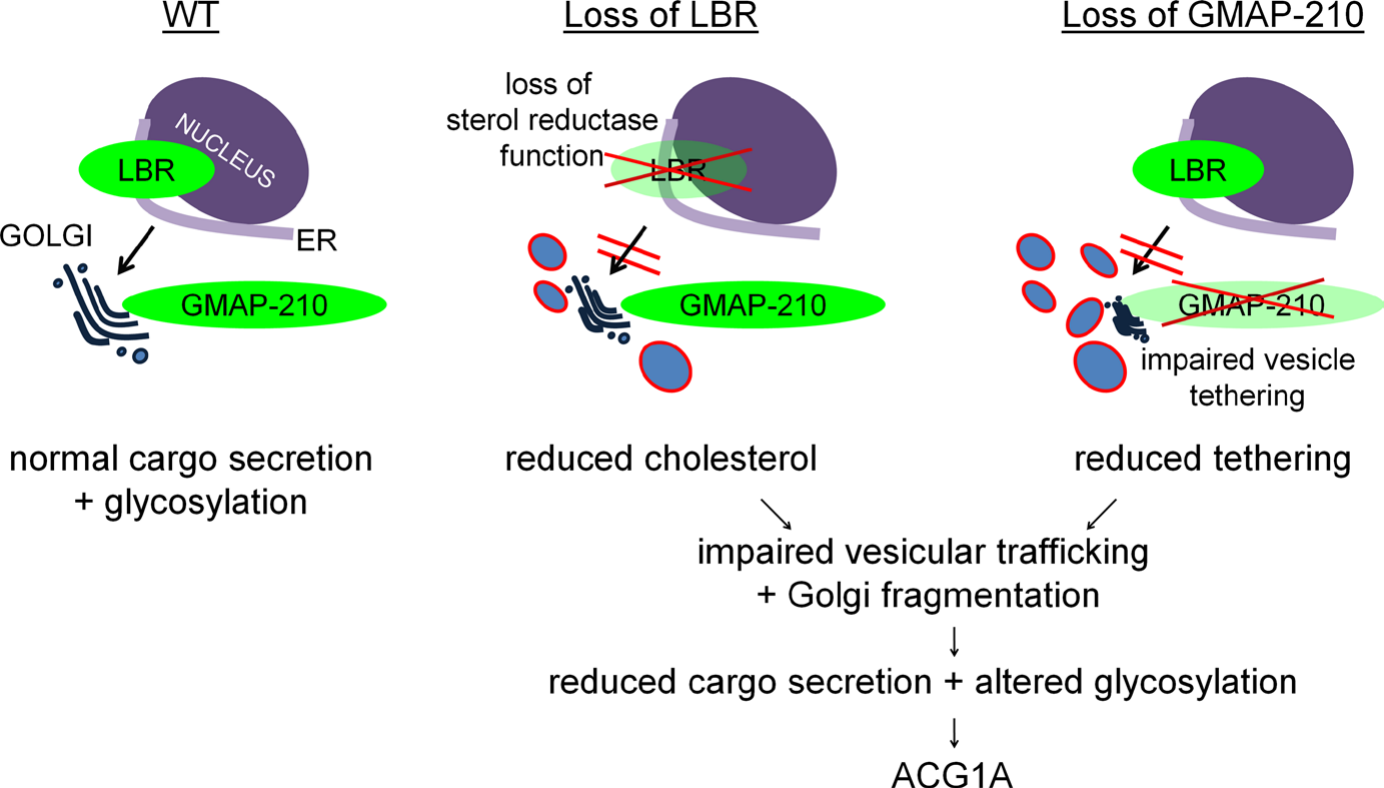
LBR and GMAP-210 deficiency impair vesicular transport in the early secretory pathway and cause defective glycoprocessing by the Golgi apparatus. Left: Wild-type cells. Middle: LBR localizes to the nuclear envelope and to the endoplasmic reticulum (ER), functioning in lamin/chromatin-binding and as a sterol reductase. Loss of LBR impairs de novo synthesis of cholesterol and causes disruption of cholesterol-sensitive ER/Golgi trafficking, resulting in defective posttranslational glycan processing and Golgi vesiculation. Right: GMAP-210 is a tether for transport vesicles at the *cis*-Golgi and is required to maintain Golgi structure. Loss of GMAP-210 also leads to disruption of ER/Golgi trafficking, impaired glycan processing, and Golgi vesiculation. Thus, both lesions converge on Golgi organization and function in achondrogenesis 1A (ACG1A).

In summary, we conclude that recessive loss-of-function mutations in *LBR* and in *TRIP11* cause a common structural and functional defect of the Golgi complex (Figure 9). As chondrogenesis and ossification critically depend on the synthesis of numerous highly glycosylated proteins, such as the extracellular matrix (ECM) proteoglycans, our findings likely represent the shared pathogenic basis of skeletal diseases caused by LBR or GMAP-210 deficiency.

## Discussion

Ever since its initial discovery as a lamin-binding nuclear receptor almost 30 years ago (34), LBR has remained an enigmatic protein. This is primarily due to the highly distinct and seemingly incompatible properties of LBR as a structural component of the inner nuclear membrane and an enzyme with specialized tasks in the biosynthesis of sterols (3, 49). Another reason for the unresolved physiological role of LBR is the lack of a genuine in vivo model that allows deduction of normal function from the knockout phenotype. To date, no animal model faithfully recapitulates human developmental anomalies associated with LBR inactivation (14–16). Recessive loss-of-function mutations of LBR are known to cause GRBGD, a prenatally lethal chondrodysplasia with recognizable radiographic features (1, 50). The most discriminative individual findings in GRBGD include the “moth-eaten” appearance of scapular and pelvic bones and ectopic calcifications of cartilage and connective tissues that, in spite of some phenotypic variability, were present in all 4 confirmed cases to date (50). GRBGD is considered to be the null phenotype of *LBR* mutations, whereas variants that are predicted to retain residual functions cause milder skeletal phenotypes (Supplemental Table 1) (6, 7). However, only few studies have characterized the effect of disease-associated mutation on *LBR* in detail (8, 12, 13), as GRBGD is an extremely rare disease and patient-derived materials are scarce. GRBGD-associated *LBR* mutations may cause alterations of neutrophil granulocyte nuclear morphology in healthy heterozygous carriers (PHA) and a similar phenotype was also seen in *Lbr*-mutant mice (15, 16). Yet, PHA is not seen in all GRBGD carriers and it remains an open question whether the *LBR*-associated nuclear lobulation defects of hematopoietic cells are due to haploinsufficiency, or caused by a dominant-negative effect of some LBR variants (5, 51). As the C14 sterol reductase activity of LBR is redundant in *Lbr*-knockout mice (14), both dominant and recessive LBR-associated diseases, as well as harmless PHA, were proposed to result from structural defects of the nuclear envelope and were classified as envelopathies (4). However, this notion remains controversial and has recently been challenged by a comprehensive study of GRBGD-causing mutations that demonstrated a nonredundant metabolic function of LBR for cholesterol biogenesis in human cells (12, 13, 49). Significantly, all studied disease-associated variants had no, or severely reduced, C14 reductase activity and were unable to rescue the cells’ dependency on exogenous cholesterol supply. The authors of this study further uncovered that truncated LBR protein variants lacking the carboxy-terminal enzymatic domain are actively eliminated at the nuclear envelope by a novel protein degradation pathway (12). Although these findings are highly significant and imply that GRBGD is in fact a metabolic disorder of cholesterol biosynthesis, the pathogenesis of developmental anomalies in LBR-associated diseases remains unknown.

Using WES, we unexpectedly identified a homozygous c.[366+1G>T] *LBR* splice site mutation in a fetus with the unambiguous clinical-radiographic diagnosis of ACG1A; all distinctive signs of GRBGD were missing in our case (1, 50). Prior to unbiased disease gene identification by WES trio analysis, the only known molecular cause of ACG1A and possible differential diagnoses had been excluded by Sanger sequencing. Molecular characterization of *LBR* in patient-derived primary cells proved a fully penetrant missplice of exon 3, resulting in a frameshift and early termination codon in the mutant ORF (LBR p.E112Sfs*39). *LBR* mRNA was barely detectable by real-time quantitative RT-PCR, compatible with NMD (27). Accordingly, both Western blotting and immunofluorescence studies demonstrated that fetal cells were completely devoid of any LBR protein; hence, the ACG1A case described here represents to our knowledge the first experimentally proven LBR-null phenotype.

Why does a complete loss of LBR result in a disease other than GRBGD? Comparing the known mutational spectrum of GRBGD (Human Gene Mutation Database) (52) with our case offers a straightforward explanation; homozygous GRBGD-associated truncations or missense mutations are all located downstream of exon 11, which preserves the N-terminal nucleoplasmic domain of LBR (encoded by exons 2 to 5 of *LBR*, Supplemental Figure 7 and Supplemental Table 1). In the single compound heterozygous GRBGD case, an early stop variant was only found in combination with a mutation affecting the 3′ end of *LBR* (Supplemental Figure 7 and Supplemental Table 1). Thus, shortened dysfunctional, or misfolded missense full-length LBR peptides comprising the lamin-interacting domain are predicted in every known confirmed GRBGD case. Recent evidence suggests that carboxy-terminal truncations render some mutant LBR proteins metabolically unstable and lead to rapid proteasomal degradation at the inner nuclear membrane (12). In contrast, the ACG1A-associated *LBR* c.[366+1G>T] mutation disrupts all annotated protein-coding transcript variants, with the exception of a very short putative variant of 51 amino acids (Supplemental Figure 7). We therefore hypothesize that, in addition to a loss of C14 reductase activity, the evolution of the distinct GRBGD disease phenotype may depend on remnant LBR protein species. Aberrant LBR proteins are translocated and actively degraded in the nucleus, thereby causing a yet to be characterized cellular (stress) response (12).

However, the effect of splice mutations in general is context dependent and may not be fully penetrant (53). Although we did not detect any correctly spliced mRNA in ACG1A fibroblasts, the homozygous *LBR* c.[366+1G>T] mutation may leave trace amounts of functional LBR in other cell types of the fetus (e.g., in chondrocytes), which may modify the GRBGD phenotype.

LBR and GMAP-210 are proteins with distinct intracellular localizations, different functions, and no known functional or physical interaction. How can mutations in unrelated genes then result in the same phenotype? We hypothesized that the similarity of developmental defects indicated a common downstream pathology and screened patient-derived primary cells for shared morphological abnormalities by deep phenotyping. In our approach, we first focused on GMAP-210–deficient cells, as there are known subcellular abnormalities of the ER and the Golgi apparatus in GMAP-deficient mice and cells (17, 23, 28, 42). However, although there is general agreement about a Golgi localization of GMAP-210 in mammals, several functions have been proposed for the protein (21, 54, 55). In particular, there is conflicting evidence regarding GMAP-210 involvement in protein trafficking and Golgi organization (21–23, 28). Importantly, whereas the ER morphology of ACG1A-derived primary cells had a variable and so far inconclusive phenotype, depending on the underlying genetic defect, our observations confirm that GMAP-210 is indeed absolutely required for Golgi organization in humans. Golgi ultrastructure was severely disrupted in *TRIP11*-mutant cells and compartment-specific markers demonstrated that loss of GMAP-210 not only affected the *cis*-Golgi, but caused compaction of the whole organelle. Our results are in line with 2 recent studies investigating the consequences of GMAP-210 depletion in generic cell models (23, 28). Efficient knockdown of GMAP-210 caused an identical Golgi compaction phenotype in HeLa cells, with ribbon fragmentation, and a drastic reduction of cisternal number and length (28), indicating that GMAP-210 is also a nonredundant structural component of the Golgi in human cells derived from other tissues. However, the most significant discovery of our study was that all ACG1A-derived primary cells exhibited a common Golgi vesiculation phenotype that was not only previously seen in GMAP-210–depleted HeLa cells (28), but in Gmap-210–deficient mouse chondrocytes as well (17, 42). In GMAP-210–depleted human cells, Golgi vesiculation is accompanied by trafficking defects in the early secretory pathway (23). We observed equivalent secretory trafficking defects in patient-derived fibroblasts, consistent with GMAP-210 acting as a rate-limiting tether for transport vesicles at the *cis*-Golgi apparatus. Indeed, vesicle formation and turnover is sensitive to any kind of change in GMAP-210 abundance, as not only the depletion, but also the overexpression of GMAP-210 in human and insect cells causes Golgi vesiculation and a block in secretory trafficking (24, 56). Our findings support a nonredundant golgin tether function of GMAP-210 for vesicular protein trafficking in human cells that likely is of particular relevance for cell types with high secretion rates, and/or complex cargoes, like ECM proteoglycans produced by chondrocytes.

While the accumulation of transport vesicles seems a plausible consequence of losing an essential Golgi tether protein, it is not obvious why a similar phenotype should ensue from impaired LBR function. However, de novo synthesis of cholesterol is compromised in LBR-mutant cells (10, 12, 13), and it has been demonstrated that alterations in cholesterol levels can induce Golgi vesiculation (44, 57). Along these lines, several publications demonstrated a reduction of ER-to-Golgi protein transport upon intracellular cholesterol depletion by conditioned media or pharmacological inhibition of de novo biosynthesis (58, 59). Structurally, cholesterol depletion leads to dispersal of Golgi cisternae (60) and although it is less well understood how, altered cholesterol levels in intracellular compartments may have an impact on soluble *N*-ethylmaleimide-sensitive-factor attachment receptor (SNARE) localization and function, which in turn could influence secretory trafficking kinetics (61). Thus, while the exact mechanism by which cholesterol impacts upon secretory trafficking has still to be unraveled, we conclude that both LBR and GMAP-210 deficiency converge on impaired vesicular transport in the early secretory pathway, most likely due to altered vesicle-membrane interactions at the ER-Golgi interface. For LBR-associated diseases, we believe our findings are novel and infer a fundamentally different pathogenesis than currently assumed (Figure 9).

How does a secretory trafficking block of the Golgi apparatus impact on skeletal development and lead to a specific achondrogenesis phenotype? Initial results from *Trip11*-knockout mice suggested that a reduced cargo flux predominantly affects cell types and tissues of the developing skeleton that have high secretion rates for ECM proteins (17). Yet, constitutive ablation of *Trip11* results in perinatal lethality, so more subtle or later disease manifestations in other tissues may have been missed. Based on recent results from conditional inactivation in mice, a limited role of Gmap-210 for cargoes exclusively secreted by chondrocytes was proposed (42). In keeping with previous results obtained in generic cell models, our findings support a broader role of GMAP-210 for secretory trafficking in humans (23, 28). Consistent with a generally impaired transit of secretory proteins through the Golgi, our findings confirm a severe glycosylation defect of Golgi-processed cargo in both *TRIP11*- and *LBR*-mutant primary human fibroblasts. Thus, incorrectly modified proteins will at least partially contribute to abnormal differentiation and ossification of skeletal tissues seen in ACG1A. This view is supported by the fact that a mere deficiency in proteoglycan modification causes a phenotypically overlapping lethal chondrodysplasia, ACG1B (62). In keeping with the ACG1A mouse model (17, 42), we hypothesize that the striking similarity of LBR- and GMAP-210–associated ACG1A may be rather based on aberrant posttranslational processing of a limited number of secreted developmental regulators with key importance for early chondrogenesis, but this clearly awaits further elucidation. We further propose that a cholesterol-dependent secretory protein trafficking defect is an underlying pathogenic mechanism that causes developmental anomalies in LBR-associated disorders. This also carries implications for other disorders of the CDP spectrum that are caused by enzyme deficiencies in the sterol biosynthesis pathway (11). Of note, skeletal defects in X-linked chondrodysplasia punctata 2 (CDPX2, MIM 3022960), due to loss-of-function mutations in the gene encoding delta(8)-delta(7)sterol isomerase emopamil-binding protein (*EBP*), may be highly similar to GRBGD (29, 63), which again might point to a common Golgi-related pathogenesis in a genetically heterogeneous disease group. Another important inference of our observations is that impaired vesicular trafficking in the secretory pathway and defective glycoprocessing may be amenable to intervention by cholesterol loading, or other pharmacological strategies to influence intracellular lipid composition. Targeting intracellular cholesterol transport mechanisms has already proved to be a successful therapeutic strategy to modify clinical manifestations in other disease contexts (58, 64).

In conclusion, we demonstrate here that mutations in *LBR* or *TRIP11* cause a common clinical and cellular phenotype that is based on impaired secretory trafficking and aberrant glycan processing by the Golgi apparatus. A glycosylation deficit is potentially central to ACG1A skeletal pathophysiology, giving reason to pursue therapeutic strategies in the future to restore normal glycosylation. Our results emphasize the importance of the Golgi complex for the developing skeleton and imply a Golgi-based disease mechanism for GRBGD and a number of related chondrodysplasias with until now unresolved pathophysiology. Further elucidation of the molecular circuits of sterol metabolism, vesicular transport, and protein modification in ACG1A and GRBGD promises exclusive insight into basic cellular mechanisms.

## Methods

### Mutation analysis.

DNA was obtained from fibroblasts by standard extraction procedures (Qiagen). For the genomic analysis and mutation detection, exons including intron-exon boundaries of *TRIP11* and *LBR* were amplified by PCR and analyzed on an ABI3130xl capillary sequencer (Life Technologies) as described previously (8, 17). Bioinformatics allele frequency analysis was based on an in-house exome database, the NHLBI Exome Sequencing Project (http://evs.gs.washington.edu/EVS/), the 1000 Genomes Project (http://www.internationalgenome.org/), and the dbSNP database (https://www.ncbi.nlm.nih.gov/projects/SNP/).

### WES.

Exome sequences were enriched from DNA using the SureSelect Exome Enrichment kit V4 (Agilent Technologies). Sequencing was performed on a 5500xl SOLiD System (Life Technologies), producing 75-bp single-end reads. For subsequent bioinformatics analysis, an in-house pipeline based on GATK best practice was used (65). Alignments were generated by the proprietary Lifescope software (66). Variant files were annotated using the variant effect predictor software (67) and processed using GEMINI (68), with filter parameters set for allele frequencies of ≤1% in the 1000 Genomes Project, an impact severity classification of medium or high, and a recessive inheritance pattern.

### Cell culture.

Human fibroblasts were derived from skin biopsies and grown at 37°C and 5% CO_2_ in Dulbecco’s modified Eagle Medium (DMEM) supplemented with 10% (vol/vol) fetal bovine serum (FBS), 1 mM L-glutamine, 1% nonessential amino acids (NEAAs, Thermo Fisher Scientific; alanine, asparagine, aspartic acid, glycine, glutamic acid, proline, and serine at 0.1 mM final concentration), 100 IU/ml penicillin and 100 μg/ml streptomycin (1% [vol/vol] PenStrep, Thermo Fisher Scientific).

### Plasmids and transfection.

ACG1A_1 fibroblasts were seeded on 10-cm dishes at 80% confluence the day before transfection in complete medium lacking antibiotics. After 24 hours, 1 × 10^6^ cells were cotransfected in 200 μl electroporation buffer (Bio-Rad, Gene Pulser buffer, 165-2676) with 2 μg pEGFP-N1 (Clontech) and 20 μg of pCDNA3.1/V5-His TOPO vector (Invitrogen) or of the respective expression plasmids LBR WT, LBR N547D, and LBR R583Q (12) (a gift from Pei-Ling Tsai, Yale University, New Haven, Connecticut, USA) using a Gene Pulser Xcell electroporation system (Bio-Rad) with a preset protocol for primary fibroblasts according to the manufacturer’s recommendations. Green-fluorescent cells were sorted on a fluorescence-activated cell sorter (FACS Aria Fusion, Becton Dickinson) 24 hours after transfection; 200,000 EGFP-positive ACG1A_1 cells were collected for each transfectant, plated in 6-well plates, and cultured for an additional 72 hours in complete growth medium before subsequent analysis.

### Quantification of cell viability and proliferation.

Triplicates of controls and patient-derived fibroblasts were seeded either on 96-well plates (5,000 cells/well), or 12-well plates (30,000 cells/well) and grown in DMEM supplemented with 10% (vol/vol) lipoprotein-depleted FBS (S5394, Sigma-Aldrich), 1 mM L-glutamine, 1% NEAAs, and 1% penicillin-streptomycin mix, or lipid-restrictive medium supplemented with SyntheChol NS0 Supplement (S5442, Sigma-Aldrich; 1:1,000 final dilution, corresponding to 6.5 μM cholesterol in aqueous solution). Day 0 values were determined 4 hours after plating. Supernatants were then removed and adherent cells quantified by crystal violet on a Tecan plate reader at 590 nm as described previously (69). Numbers of viable cells for a crystal violet dye reference curve and for those grown in 12-well plates were automatically counted after trypsinization by a Muse cell analyzer (Merck Millipore) as described by the manufacturer.

### Cholesterol depletion of fibroblasts.

Cells were seeded on 3.5-cm dishes and grown in complete medium until they reached 80% confluence and then incubated for 48 hours in serum-free DMEM supplemented with 2.5 mM MβCD or 2.5 mM MβCD:cholesterol–saturated complexes. MβCD:cholesterol complexes were prepared as described previously (30). Briefly, cholesterol was dissolved in a mixture of chloroform and methanol (2:1, vol/vol) to make a 50 mg/ml stock solution. Sixteen microliters of the solution was put into a glass vial and nitrogen gas was used to evaporate the solvent, creating a thin film of cholesterol, which was dissolved in 2.5 mM MβCD prepared in serum-free DMEM. The solution was further sonicated for 30 minutes in an ultrasonic bath, incubated overnight at 37°C with agitation, and filtered through a 0.22-μm syringe filter prior to use. Images were collected on an Olympus IX83 inverted microscope using a 20×/0.45 LUC PlanFL N objective with phase-contrast and captured using an Orca ER camera (Hamamatsu) through CellSens software (Olympus). Images were then processed and analyzed using ImageJ (NIH).

### Sterol analysis of cultured cells.

Human fibroblasts were grown at 37°C and 5% CO_2_ for 5 days in 10% (vol/vol) lipoprotein-deficient serum (S5394, Sigma-Aldrich) supplemented with 1% NEAAs and 1% penicillin-streptomycin mix. Cells were harvested by centrifugation, washed 3 times with PBS, sonicated, lysed in TNE buffer (50 mM Tris-HCl [pH 7.5], 20 mM NaCl, 0.1 mM EDTA) and extracted with methanol/chloroform. After silylation, alkaline hydrolysis and derivatization with *N*,*O*-bis(trimethylsilyl)trifluoroacetamide (BSTFA), samples were analyzed by GC/MS using a triple quadrupole mass spectrometer (Agilent 6460 QQQ); 5β-cholestan-3α-ol was used as internal standard.

### Pulse-chase analysis of protein secretion.

Analysis of protein secretion was performed using a pulse-chase approach as described previously (45). Cells were seeded onto 3.5-cm dishes and grown until they reached 95% confluence. Cells were starved for 1 hour in methionine- and cysteine-free DMEM (Invitrogen) supplemented with 10% (vol/vol) FBS and 1 mM L-glutamine and pulsed for 20 minutes with fresh starvation medium containing 50 μCi/ml [^35^S]Met and [^35^S]Cys protein-labeling mix (PerkinElmer). After washing with PBS, cells were incubated in chase medium (DMEM, 10 mM HEPES pH 7.4, 1 mM methionine, 1 mM cysteine, 100 μg/ml bovine serum albumin) for 0, 30, or 60 minutes. Proteins secreted to the medium were precipitated using trichloroacetic acid (TCA), while cells were lysed using HMNT lysis buffer. Cells and medium fractions were subjected to SDS-PAGE, gels were fixed for 10 minutes in 20% (vol/vol) methanol and 10% (vol/vol) acetic acid, and dried for 2 hours at 65°C using a gel dryer (Bio-Rad). Dried gels were exposed to phosphorimaging plates and developed after 10 days using a fluorescent image analyzer (FLA 7000, Fujifilm). Signals from radiolabeled proteins were quantified using AIDA software (Elysia-Raytest).

### RNA extraction, reverse transcription PCR, and quantitative PCR.

Total RNA from fibroblasts and cell lines was extracted with TRIzol (Invitrogen), followed by DNase I treatment (Roche) and column purification (Qiagen). RNA was quantified by spectrophotometry (Nanodrop ND-8000, peqlab), and cDNA was synthesized from 1 μg total RNA with Superscript III reverse transcriptase (Invitrogen) and oligo dT16 primers. Quantitative PCR was performed on a CFX384Touch Real-Time PCR System (Bio-Rad). Delta cycle threshold (Ct) relative quantification, PCR efficiency correction, and multiple reference gene normalization (*UBC*, *TBP*, *HMBS*, and *PDHA*) were calculated with qBase (Bio-Rad). Oligonucleotides for *TRIP11* and *LBR* were designed using the NCBI Primer Blast software (https://www.ncbi.nlm.nih.gov/tools/primer-blast/). Primer specificity was verified by agarose gel electrophoresis, Sanger sequencing of PCR products, and melting curve analysis.

### Antibodies.

The following antibodies were used: rabbit anti-LBR (ProteinTech, 12398-1-AP), goat anti–lamin B (C-20; Santa Cruz Biotechnology), mouse anti–ERGIC-53 (G1/93; Enzo Life Sciences), rabbit anti–GMAP-210 (HPA002570, Sigma-Aldrich), mouse anti-p230 (clone 15; BD Biosciences), rabbit anti-PDI (SPA-890; Stressgen Biotechnologies), rabbit anti-Sec13 (Rainer Pepperkok, European Molecular Biology Laboratory, Heidelberg, Germany), sheep anti-ZFPL1 (70), rabbit anti-giantin (Manfred Renz, Institute of Immunology and Molecular Genetics, Karlsruhe, Germany), mouse anti-GAPDH (G-9; Santa Cruz Biotechnology), rabbit anti–LAMP-1 (D2D11, Cell Signaling Technology), and anti-LAMP2 (H4B4; Developmental Studies Hybridoma Bank, University of Iowa, Iowa City, Iowa, USA). Alexa 488–, 546–, 594–, and 647–conjugated secondary antibodies were from Molecular Probes (Thermo Fisher Scientific). Horseradish peroxidase–conjugated secondary antibodies were from Sigma-Aldrich.

### Immunoblotting.

Fibroblasts were lysed using either radioimmunoprecipitation assay buffer (RIPA; 20 mM Tris-HCl [pH 7.5], 150 mM NaCl, 1 mM Na_2_EDTA, 1 mM EGTA, 1% NP-40, 1% sodium deoxycholate, 2.5 mM sodium pyrophosphate, and 1 mM β-glycerophosphate) supplemented with dithiothreitol (DTT, Bio-Rad), benzonase (Merck Millipore), protease inhibitor cocktail (Calbiochem), PhosStop (Roche), or HMNT lysis buffer supplemented with protease inhibitor cocktail; 30 μg of cell lysates was separated by SDS-PAGE. Proteins were then transferred onto nitrocellulose membranes (Amersham Hybond 0.45 NC, GE Healthcare) using a wet transfer system. The efficiency of transfer was assessed by Ponceau S stain (Sigma-Aldrich). Membranes were blocked by incubation in PBST buffer (PBS with 0.1% Tween 20) supplemented with 5% skim milk for 1 hour at room temperature (RT) and incubated with primary antibody solution (5% skim milk in PBST) overnight at 4°C. Membranes were washed 3 times in PBST buffer, for 5 minutes each at RT (all subsequent steps at RT), and incubated with secondary antibody solution (5% skim milk in PBST) for 1 hour. Membranes were washed 3 times in PBST buffer for 5 minutes and once in PBS for 5 minutes. The immunoreaction was detected with SuperSignal West Pico ECL chemiluminescent substrate (Thermo Fisher Scientific) using a Bio-Rad Chemidoc MP Imager. Data were analyzed using Image Lab software (Bio-Rad).

### Glycan processing analysis of fibroblasts.

For the analysis of cholesterol-dependent protein glycosylation, cells were seeded on 3.5-cm dishes and grown in complete medium until they reached 80% confluence and then incubated for 24 or 48 hours in serum-free DMEM supplemented with 1.25 mM MβCD. Cells were lysed using HMNT lysis buffer (20 mM HEPES-KOH pH 7.4, 5 mM MgCl_2_, 0.1 M NaCl, 0.5% [wt/vol] Triton X-100) supplemented with protease inhibitor cocktail (Calbiochem) and 30 μg of cell lyses was subjected to SDS-PAGE, as described above, and blotted with anti-LAMP2 antibody.

### Fluorescence microscopy.

Fibroblasts were seeded on glass coverslips and grown to 90% confluence. Cells were washed twice with PBS, fixed with 3% (wt/vol) paraformaldehyde (PFA; Sigma-Aldrich) in PBS for 25 minutes at RT. Cells were then washed with PBS and excess PFA was quenched with glycine. Cells were permeabilized by 4-minute incubation in 0.1% (wt/vol) Triton X-100 in PBS. For ER morphology analysis, cells were fixed for 20 minutes at RT in a fixative solution composed of 3% (wt/vol) PFA and 0.025% (wt/vol) glutaraldehyde (GA; electron microscopy-grade, Sigma-Aldrich). Cells were then washed 3 times with PBS. PFA-GA was quenched by washing 3 times in freshly prepared 1 mg/ml NaBH_4_ solution in PBS for 5 minutes at RT followed by 3 washes with PBS. Cells were then permeabilized by 4-minute incubation in 0.1% (wt/vol) Triton X-100 and 0.05% (wt/vol) SDS in PBS. Cells were incubated with primary antibody diluted in PBS for 1 hour at RT and incubated 3 times with PBS for 5 minutes. Then coverslips were incubated for 1 hour with secondary antibody diluted in PBS, sometimes together with 100 ng/ml Hoechst 33342 dye (Thermo Fisher Scientific) to stain DNA, and incubated 3 times with PBS for 5 minutes and twice in ddH_2_0 for 5 minutes. Coverslips were dried before mounting in Mowiol 4-88. Images were acquired using an Olympus BX60 upright microscope equipped with a MicroMax cooled, slow-scan CCD camera (Princeton Instruments) driven by Metaview software (University Imaging Corporation). Images were processed using ImageJ software.

### Electron microscopy.

Cells were grown on 10-cm dishes until they reached 80% confluence. The samples were fixed with 4% PFA plus 2.5% GA in 0.1 M HEPES buffer (pH 7.2). Fixed cells were scraped and centrifuged for 10 minutes at 5,200 *g*. They were then postfixed with 1% osmium tetroxide plus 1.5% potassium ferrocyanide in 0.1 M cacodylate buffer (pH 7.2) for 1 hour and in 1% uranyl acetate in water for 1 hour. The postfixed samples were dehydrated in ethanol series infiltrated with Low Viscosity resin (TAAB) and polymerized for 24 hours at 60°C. Sections (70 nm) were cut with an Ultracut ultramicrotome (Reichert) and observed with an FEI Tecnai 12 Biotwin microscope at 100 kV accelerating voltage. Images were obtained with a Gatan Orius SC1000 CCD camera. Quantification of cells with large vesicular profiles, defined as peri-Golgi circular profiles with a diameter of >100 nm, were conducted manually in a blind manner.

### Statistics.

Statistical analyses were conducted using GraphPad Prism. D’Agostino-Pearson and Shapiro-Wilk tests were used for comparison of the distribution of data with a Gaussian distribution. Depending on the result, a Mann-Whitney test, 1-way or 2-way ANOVA with Bonferroni’s correction for multiple comparisons was performed. The number of cells with large vesicular profiles was compared using a χ^2^ test. Amounts of secreted proteins were compared using 1-way ANOVA followed by Dunnett’s multiple comparisons test. Statistical significance cutoffs were set as follows: *P ≤ 0.05, **P < 0.01, ***P < 0.001, and ****P < 0.0001.

### Study approval.

All individuals in our study were recruited by physician-initiated referral. The study was conducted in accordance with the Declaration of Helsinki protocols and approved by the institutional ethics review board of Freiburg University Hospital, Germany (Ethik-Kommission der Albert-Ludwigs-Universität Freiburg Nr. 349/13). Written informed consent for skin biopsies and molecular studies was obtained from the parents, or legal guardians of included cases in accordance with current German law (GenDG). Control samples and primary cells were collected from matched healthy individuals under the same criteria and regulations.

## Author contributions

EL and ML designed the research studies. AW, TMW, and JCS conducted experiments and acquired data. AW, TMW, JCS, SB, JS, AH, AK, ME, EL, and ML contributed and analyzed data. AW and EL wrote the manuscript. AW, TMW, EL, and ML edited the manuscript. All other co-authors read, commented on, and approved the manuscript.

## Supplementary Material

Supplemental data

## Figures and Tables

**Table 1:**
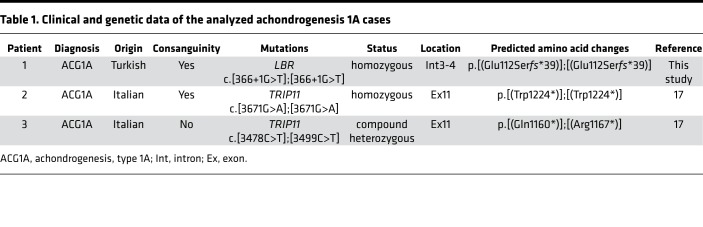
Clinical and genetic data of the analyzed achondrogenesis 1A cases

## Version 1 12/06/2018

Electronic publication
