# Supplementary material for: A common pathomechanism in GMAP-210– and LBR-related diseases
Source: JCI Insight. 2018 Dec 6;3(23):e121150. doi: 10.1172/jci.insight.121150 (PMC6328090; doi:10.1172/jci.insight.121150)
Supplement: Supplemental data [file jciinsight-3-121150-s099.pdf]

Supplemental information

A common pathomechanism in GMAP-210 and LBR-related diseases.

Anika Wehrle, Tomasz M. Witkos, Judith Schneider, Anselm Hoppmann, Sidney Behringer, Anna Köttgen, Mariet Elting, Jürgen Spranger, Martin Lowe, Ekkehart Lausch.

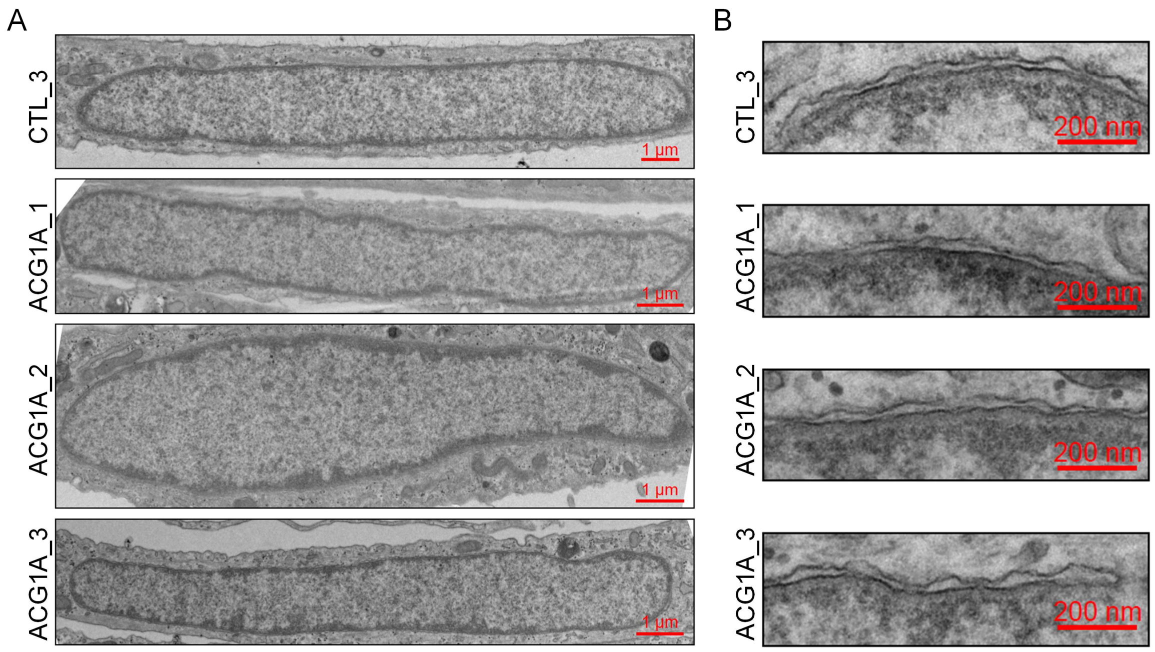

Supplemental Figure 1: Transmission electron microscopy of the nucleus in achondrogenesis 1A (ACG1A). (A) Representative sections through complete nuclei to assess shape and structure; scale bar=1µm. **(B)** Zoomed images focusing on spacing between the inner and outer membrane of the nuclear envelope in controls (CTL_3) and patient fibroblasts (ACG1A_1 to 3); scale bar=200nm.

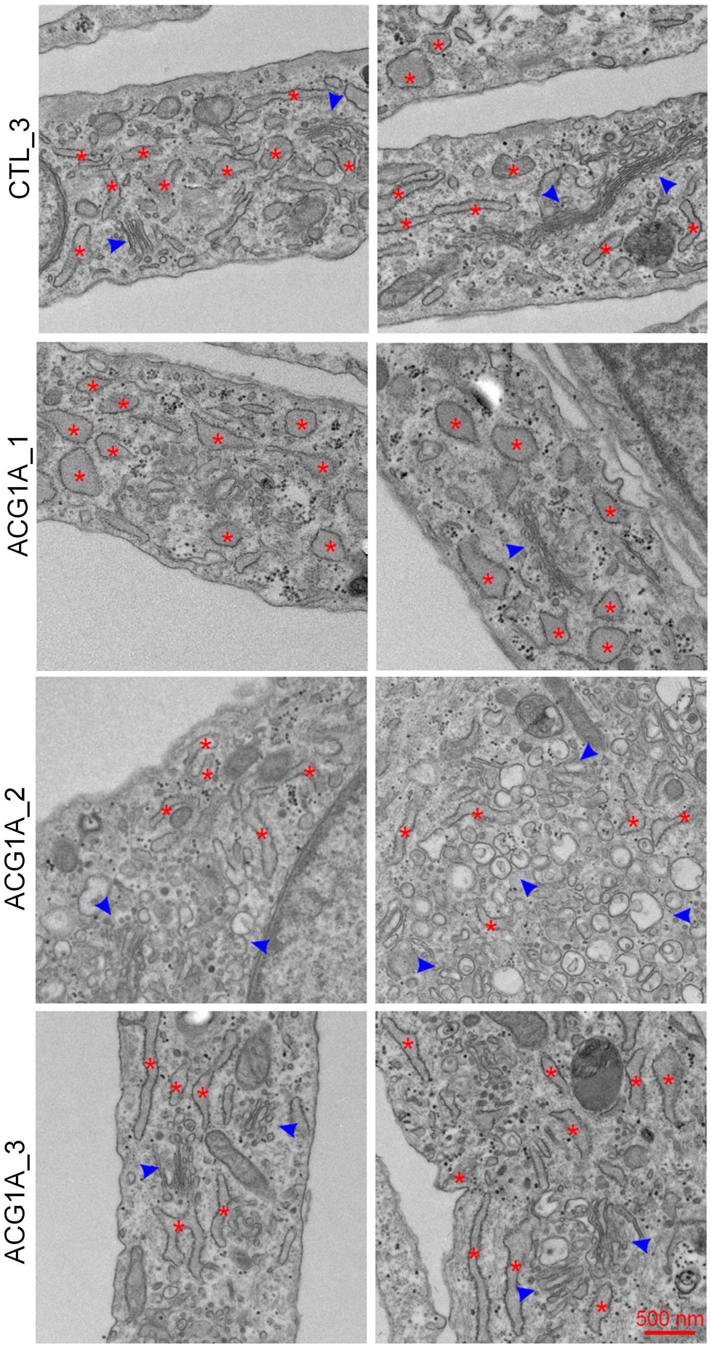

Supplemental Figure 2: Transmission electron microscopy of the endoplasmic reticulum (ER) and the Golgi apparatus in achondrogenesis 1A (ACG1A). Representative sections through the ER (red asterisk) and the Golgi region (blue arrow heads) of control (CTL_3) and patient fibroblasts (ACG1A_1 to 3); scale bar=500nm.

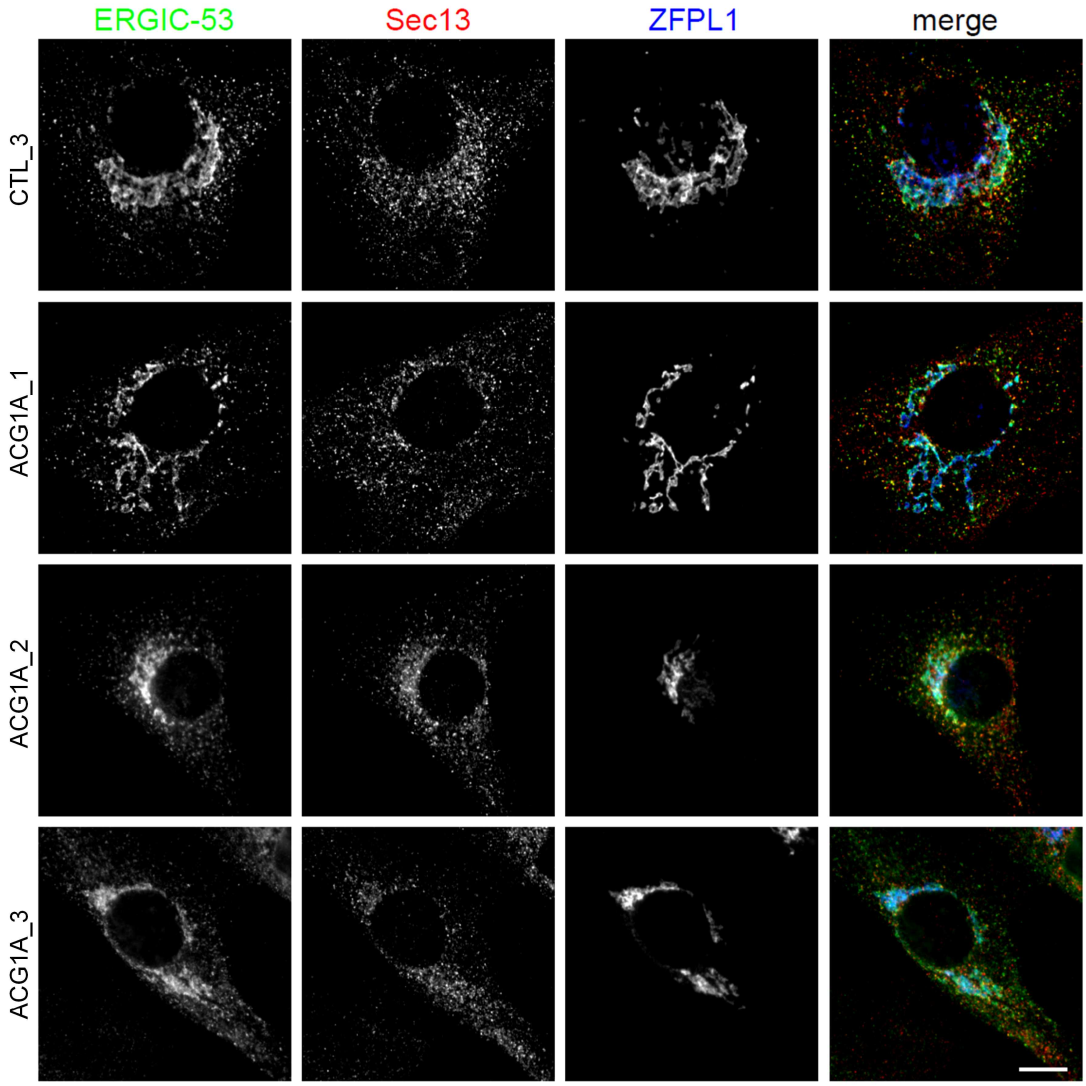

Supplemental Figure 3: Immunofluorescence microscopy of early secretory pathway compartments in achondrogenesis 1A (ACG1A). Wide-field microscopy of control (CTL_3) and patient fibroblasts (ACG1A_1 to 3) co-stained with antibodies directed against the endoplasmic reticulum (ER)-Golgi intermediate compartment (ERGIC) marker ERGIC-53, the coat protein complex II (COPII) component SEC13 that marks ER exit sites (ERES), and the *cis*-Golgi protein ZFPL1; scale bar=10µm.

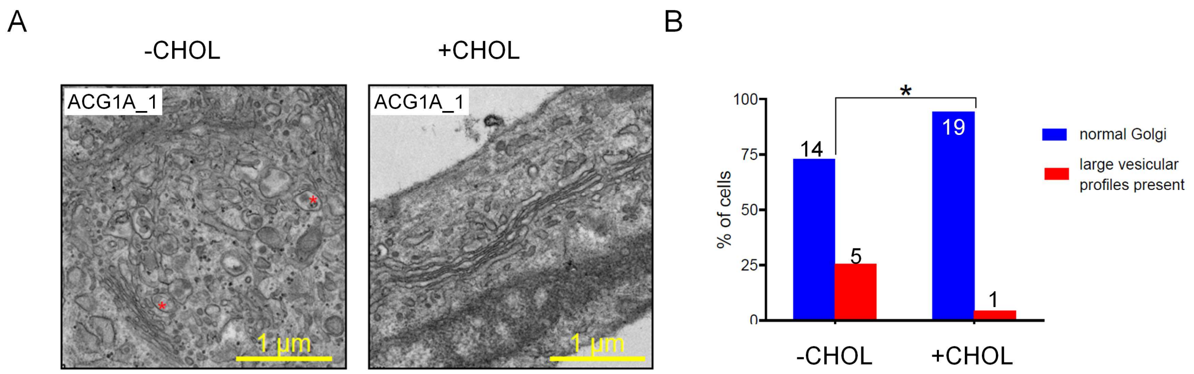

Supplemental Figure 4: Exogenous cholesterol restores normal Golgi morphology in achondrogenesis 1A (ACG1A). (A) ACG1A_1 cells were grown for five days in lipoprotein-depleted medium (-CHOL) or in the same medium supplemented with 6.5µM SyntheChol (+CHOL) and analyzed by transmission electron microscopy. Red arrowheads mark large vesicular profiles. Scale bar=1µm. **(B)** Quantification of cells with abnormal vesiculation, defined as peri-Golgi circular profiles with a diameter of >100nm (red bar) compared to cells with normal Golgi morphology (blue bar). The y-axis shows per cent of cells with the respective phenotypes, total cell numbers are indicated above the bars. The number of cells with large vesicular profiles was compared using a chi-square test; *$p<0.05$.

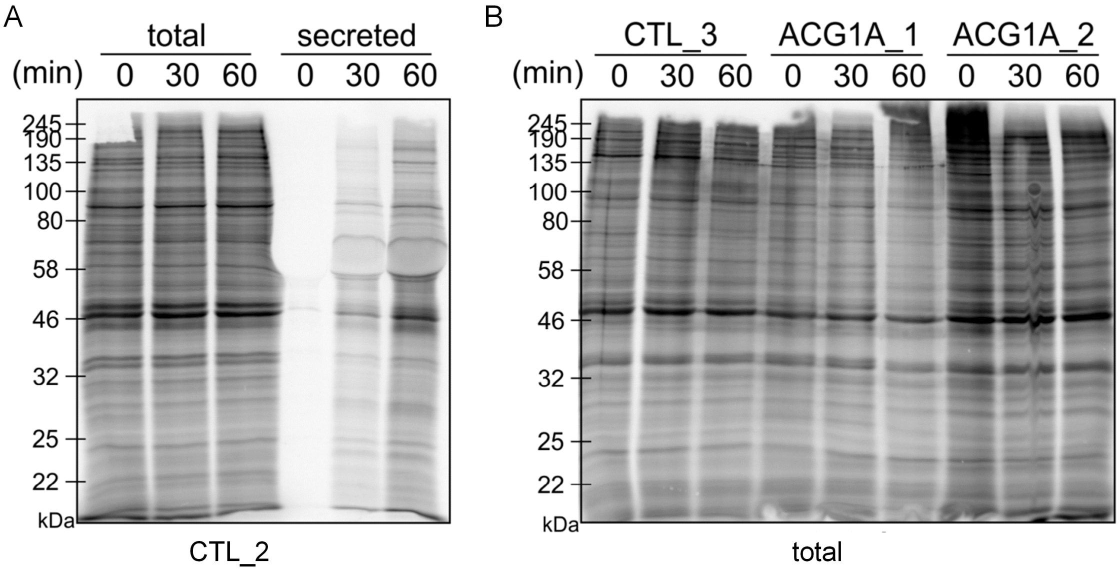

Supplemental Figure 5: Deficiency of GMAP-210 and LBR decreases secretory trafficking. Controls (CTL_2 and 3) and patient fibroblasts (ACG1A_1 and 2) were starved for 1h in methionine and cysteine-free medium and then pulsed for 20 min with fresh starvation medium containing 50µCi/ml [^{35}S]methionine and [^{35}S]cysteine protein labeling mix. Cells were chased in medium containing unlabeled methionine and cysteine for 0, 30, and 60min. Proteins secreted into the medium were precipitated by trichloroactetate (TCA) and lysed. Radiolabeled secreted proteins in TCA precipitates (‘secreted’) and total cell lysates (‘total’) were subjected to sodium dodecyl sulfate polyacrylamide gel electrophoresis and visualized by phosphorimaging. **(A)** Autoradiogram of total and secreted proteins of CTL_2, the radiolabelled proteins shown in **(B)** correspond to the secreted proteins of CTL_3, ACG1A_1 and 2 in Figure 7A.

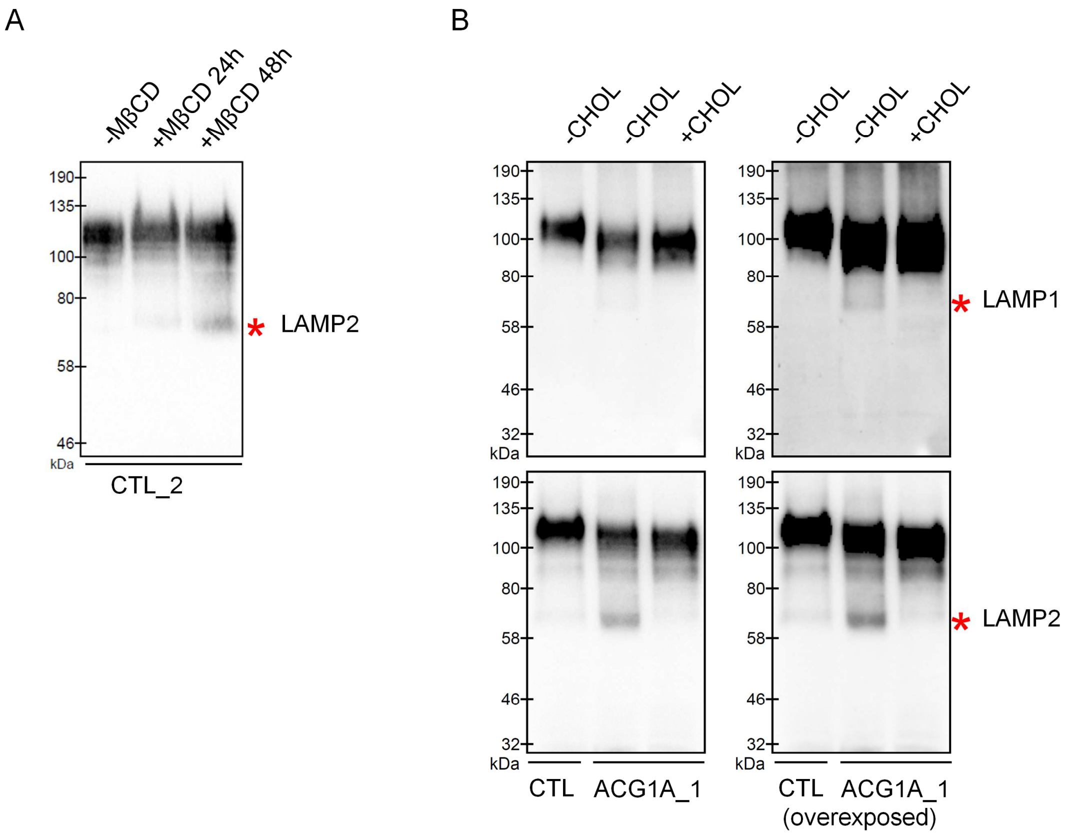

Supplemental Figure 6. Glycan processing by the Golgi apparatus depends on cellular cholesterol levels. Immunoblot analyses of LAMP1 and LAMP2 proteins in whole-cell lysates of patient and control fibroblasts. **(A)** Low molecular weight intermediate glycosylation products of LAMP2 in CTL_2 cells treated with MβCD for 24 and 48h are marked by a red asterisk. **(B)** Immunoblot analyses of LAMP1 and LAMP2 proteins in whole-cell lysates of patient and control fibroblasts grown for 5 days in lipoprotein-depleted medium (-CHOL) or with 6.5µM SyntheChol supplement (+CHOL). Right: Membranes were overexposed to detect weaker signals. Low molecular weight intermediate glycosylation products of LAMP1 and LAMP2 are marked by a red asterisk.

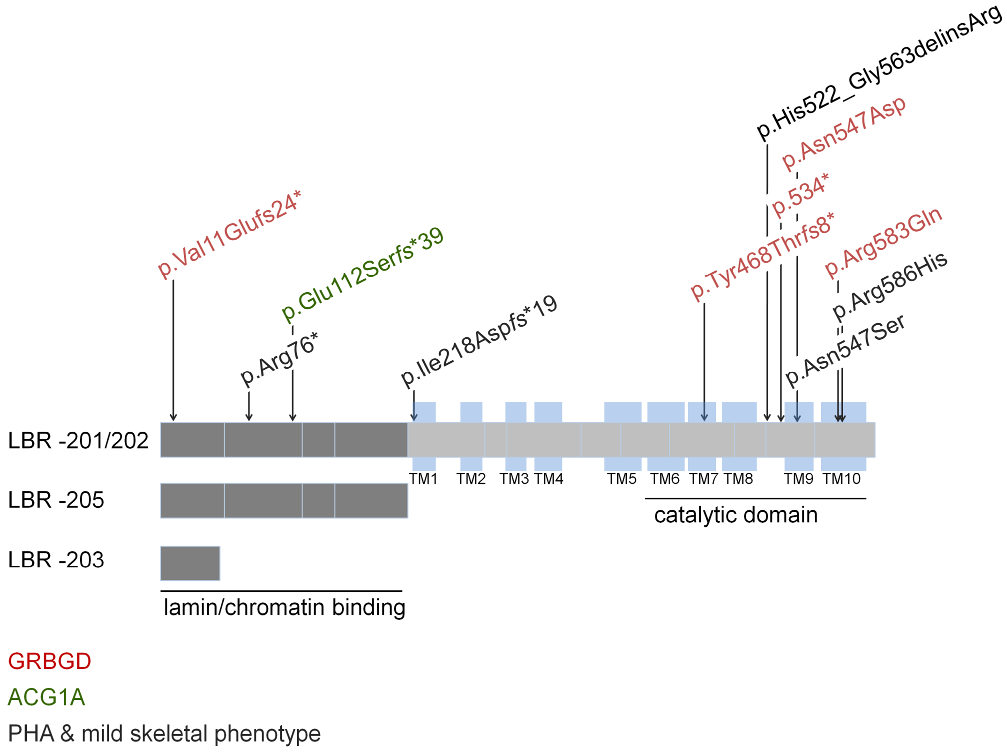

Supplemental Figure 7: *LBR* mutations cause a spectrum of skeletal phenotypes. Schematic representation of different protein isoforms encoded by the *LBR* locus (according to ensembl; http://www.ensembl.org). Boxes indicate individual exons. Arrows mark the position of each mutation in the protein (see also Supplementary Table 1). LBR-201/202 contains ten transmembrane segments (TM1–10). The catalytic domain comprises the carboxy-terminal half (containing TM6–10). Mutations associated with Greenberg dysplasis (GRBGD, shown in red) are either missense or truncating mutations causing perturbed enzymatic activity or metabolic instability of the LBR protein; homozygous mutations inactivate the sterol reductase domain, compound heterozygous mutations combine early stop with distal missense changes. Compound *LBR* missense and splice site mutations resulting in Pelger-Huët anomaly (PHA) and milder skeletal phenotypes are shown in black. The homozygous *LBR* mutation causing achondrogenesis 1A (ACG1A) is depicted in green.

Case	Phenotype	Mutations (gene and protein)	Type of mutation	Status	Location	Experimentally observed effects for individual mutations	Sterol analysis	Reference
1	PHA+ GRBGD	c.1599_1605delTCTTCTAinsCTAGAAG; p.534*	nonsense	homozygous	Ex13	- heterozygous PHA<br>- truncation of LBR<br>- degradation at inner nuclear membrane;<br>- induction of vacuole-like structures when overexpressed in U2OS cells	increased levels of cholesta-8,14-dien-3β-ol in skin fibroblasts	1, 2, 3
2	GRBGD	c.1639 A>G; p.Asn547Asp	missense	homozygous	Ex13	- heterozygous no PHA<br>- perturbed enzymatic activity of LBR by reduced affinity for NADPH	n.a.	1, 4
3	GRBGD	c.1402delT; p.Tyr468Thr*fs*8*	frameshift/stop	homozygous	Ex11	- PHA?<br>- truncation of LBR<br>- degradation at inner nuclear membrane	n.a.	1, 5
4	PHA+ GRBGD	c.32_35delTGGT; p.Val11Glu*fs*24*	frameshift/stop	compound heterozygous	Ex2	- PHA	abnormal sterol metabolite 5α-cholesta-8,14-dien-3β-ol in muscle tissue	1, 5
		c.1748G>A; p.Arg583Gln	missense		Ex14	- No PHA<br>- perturbed enzymatic activity of LBR by reduced affinity for NADPH		
5	PHA+ spondylo-metaphyseal dysplasia,	c.226C>T; p.Arg76*	nonsense	compound heterozygous	Ex3	n.a.	increased levels of cholesta-8,14-dien-3β-ol	6
		c.1640A>G; p.Asn547Ser	missense		Ex13			

6	PHA+ mild skeletal phenotype	c.651_653delinsTGATGAGAAA; p.Ile218Asp*fs**19	frameshift/stop	compound heterozygous	Ex6	n.a.	increased levels of cholesta-8,14-dien-3β-ol	7
		c.1757G>A; p.Arg586His	missense		Ex14			
7	PHA+ mild skeletal phenotype+ additional findings	c.1688-10_1688-5delTTATCC; p.His522_Gly563delinsArg	splice site mutation	homozygous	Int13-14[A]	-homozygous PHA -loss of exon 13. No mutant protein in lymphoblastoid cells; small amount of wild-type LBR; -inducing vacuole-like structures when overexpressed in U2OS cells	n.a.	3, 8
8	**ACG1A**	c.366+1G>T; p.Glu112Ser*fs**39	splice site mutation	homozygous	Int3-4	-partial loss of exon 3 -no mutant protein in fibroblasts.	increased levels of cholesta-8,14-dien-3β-ol	this work

Supplemental Table 1: Overview of *LBR* mutations and associated skeletal phenotypes. PHA: Pelger-Huët anomaly; GRBGD: Greenberg dysplasia; Ex: exon; Int: intron; n.a.: not available; [A] erroneously annotated as intron 12-13 in the original publication.

Supplemental references

1. Tsai PL, Zhao C, Turner E, Schlieker C. The Lamin B receptor is essential for cholesterol synthesis and perturbed by disease-causing mutations. *Elife* **5**, (2016).

2. Waterham HR*, et al.* Autosomal recessive HEM/Greenberg skeletal dysplasia is caused by 3 beta-hydroxysterol delta 14-reductase deficiency due to mutations in the lamin B receptor gene. *Am J Hum Genet* **72**, 1013-1017 (2003).

3. Zwerger M, Kolb T, Richter K, Karakesisoglou I, Herrmann H. Induction of a massive endoplasmic reticulum and perinuclear space expansion by expression of lamin B receptor mutants and the related sterol reductases TM7SF2 and DHCR7. *Mol Biol Cell* **21**, 354-368 (2010).

4. Konstantinidou A*, et al.* Pathologic, radiographic and molecular findings in three fetuses diagnosed with HEM/Greenberg skeletal dysplasia. *Prenat Diagn* **28**, 309-312 (2008).

5. Clayton P*, et al.* Mutations causing Greenberg dysplasia but not Pelger anomaly uncouple enzymatic from structural functions of a nuclear membrane protein. *Nucleus* **1**, 354-366 (2010).

6. Sobreira N*, et al.* An anadysplasia-like, spontaneously remitting spondylometaphyseal dysplasia secondary to lamin B receptor (LBR) gene mutations: further definition of the phenotypic heterogeneity of LBR-bone dysplasias. *Am J Med Genet A* **167A**, 159-163 (2015).

7. Borovik L, Modaff P, Waterham HR, Krentz AD, Pauli RM. Pelger-huet anomaly and a mild skeletal phenotype secondary to mutations in LBR. *Am J Med Genet A* **161A**, 2066-2073 (2013).

8. Hoffmann K*, et al.* Mutations in the gene encoding the lamin B receptor produce an altered nuclear morphology in granulocytes (Pelger-Huet anomaly). *Nat Genet* **31**, 410-414 (2002).
